# The role of antimicrobial resistance on long-term mortality and quality of life in critically ill patients: a prospective longitudinal 2-year study

**DOI:** 10.1186/s12955-021-01712-0

**Published:** 2021-03-03

**Authors:** Triantafyllia Koukoubani, Demosthenes Makris, Zoe Daniil, Theoniki Paraforou, Vasiliki Tsolaki, Epaminondas Zakynthinos, John Papanikolaou

**Affiliations:** 1 Department of Critical Care, General Hospital of Trikala, Thessaly, Greece; 2 Department of Critical Care, School of Medicine, University of Thessaly, University Hospital of Larissa, Biopolis, 41110 Larissa, Greece

**Keywords:** Health-related quality of life, Quality-adjusted life years, Long-term outcomes, Mortality, Patient-important outcomes, Multi-drug resistance

## Abstract

**Background:**

In the recent era, antimicrobial resistance has been identified as one of the most important threats to human health worldwide. The rapid emergence of antibiotic-resistant pathogens (ABRP) in the modern intensive care unit (ICU) also represents a “nightmare scenario” with unknown clinical consequences. In the Greek ICU, in particular, gram negative ABRPs are now considered endemic. However, the possible longitudinal impact of ABRPs on long-term outcomes of ICU patients has not yet been determined.

**Methods:**

In this two-year (January 2014-December 2015) single-centre observational longitudinal study, 351 non-neurocritical ICU patients ≥ 18 year-old were enrolled. Patients’ demographic, clinical and outcome data were prospectively collected. Quality-adjusted life years (QALY) were calculated at 6, 12, 18 and 24 months after ICU admission.

**Results:**

Fifty-eight patients developed infections due to ABRP (ABRP group), 57 due to non-ABRP (non-ABRP group), and 236 demonstrated no infection (no-infection group) while in ICU. Multiple regression analysis revealed that multiple organ dysfunction syndrome score (OR: 0.676, 95%CI 0.584–0.782; P < 0.001) and continuous renal replacement therapy (OR: 4.453, 95%CI 1.805–10.982; P = 0.001) were the only independent determinants for ABRP infections in ICU. Intra-ICU, 90-day and 2-year mortality was 27.9%, 52.4% and 61.5%, respectively. Compared to the non-ABRP and no-infection group, the ABRP group demonstrated increased intra-ICU, 90-day and 2-year mortality (P ≤ 0.022), worse 2-year survival rates in ICU patients overall and ICU survivor subset (Log-rank test, P ≤ 0.046), and poorer progress over time in 2-year QALY kinetics in ICU population overall, ICU survivor and 2-year survivor subgroups (P ≤ 0.013). ABRP group was further divided into multi-drug and extensively-drug resistant subgroups [MDR (n = 34) / XDR (n = 24), respectively]. Compared to MDR subgroup, the XDR subgroup demonstrated increased ICU, 90-day and 2-year mortality (P ≤ 0.031), but similar 90-day and 2-year QALYs (P ≥ 0.549). ABRP infections overall (HR = 1.778, 95% CI 1.166–2.711; P = 0.008), as well as XDR [HR = 1.889, 95% CI 1.075–3.320; P = 0.027) but not MDR pathogens, were independently associated with 2-year mortality, after adjusting for several covariates of critical illness.

**Conclusions:**

The present study may suggest a significant association between ABRP (especially XDR) infections in ICU and increased mortality and inability rates for a prolonged period post-discharge that requires further attention in larger-scale studies.

## Background

Intensive Care Unit (ICU) survivors may suffer tremendous changes in lifestyle post-discharge [1]. Critical illness may impair mental, physical and social health, limit patients’ mobility and independence, and damage self-confidence, sense of hope and positive outlook [2]. These devastating consequences of critical illness, although of limited “clinical relevance”, are now considered as “patient-important outcomes” (such as quality of life, functional/cognitive/neurological outcomes assessed after ICU discharge) and their role in clinical research is increasingly emphasized [3, 4]. In addition, the importance of assessing intermediate and long-term outcomes post-ICU discharge has also been encouraged [5].

Antibiotic resistant pathogens (ABRP) have been identified as one of the most important threats for the modern ICU, limiting treatment options and resulting in adverse clinical outcomes and excessive cost of care [6–8]. In the Greek ICU, in particular, Gram-negative multi-drug resistant (MDR) and extensively-drug resistant (XDR) bacteria are considered endemic [9, 10]. The impact of antimicrobial resistance on ICU mortality has been widely studied so far [9–20]; however, the putative influence of ABRP-induced ICU infections on post-ICU patient-important, intermediate and long-term outcomes remains largely undetermined so far.

The complex consequences of critical illness are usually referred to as “quality of life” indices, on the basis of several different instruments (questionnaires, phone or personal interviews, physical examinations, chart interviews, neuro-cognitive tests) which were developed in order to assess outcomes [21]. These markers reflect the individual aspect of ‘happy to live’ as a result of multidimensional perceptions of physical, psychological, and social variables [2]. An established outcome index is quality-adjusted life year (QALY) index, which receives a growing interest in ICU research, as it combines both quantity and quality of life gained as a result of ICU admission and implementation of advanced therapy [1, 22–26].

The aim of our study was to examine whether antimicrobial resistance in ICU is associated with increased long-term mortality and problematic quality of life for a prolonged time period post-ICU admission. For this purpose, ICU patients were divided into three subgroups: patients having suffered ABRP-induced ICU infection(s) (ABRP-group), non-ABRP infection(s) (non-ABRP-group) and patients who manifested no infection at all while in ICU (“no-infection” group). Survival data and QALY measurements were prospectively recorded over a two-year follow-up period.

## Material and methods

### Patient population

All consecutive adult patients admitted in the eight-bed medical-surgical ICU of a Greek regional community hospital (General Hospital of Trikala, Thessaly, Greece) between January 1, 2014, and December 31, 2015, were prospectively included. The study was approved by the Institutional Review Board and Ethics Committee of General Hospital of Trikala -ID: 123/October 15th/2013.

Written informed consent was obtained from each patient or his/her legal representative.

All patients ≥ 18 years old were considered as eligible for the study. Post-operative patients included both elective and emergency surgical patients. Exclusion criteria were: (1) Patients confined to Hospital and/or to bed prior to ICU admission, and (2) neurosurgical patients requiring advanced neurocritical support (transmission to a tertiary center). Patients who refused to cooperate or were not found at follow-ups were also excluded from the study.

Demographic data, pre-hospitalization clinical status (QALY values estimated for the last year prior to ICU admission) and comorbidities, admitting etiology, Acute Physiology and Chronic Health Evaluation II (APACHE II) on admission, clinical data including ICU infections/microbiology, treatment data including colistin administration, intra-ICU outcomes and survival data post-discharge were prospectively collected. The severity of multiple organ dysfunction syndrome (MODS) was assessed upon admission and during ICU infection(s), by using the MODS score as previously described [27]; the maximum MODS score value was analyzed for each patient.

Infections were defined according to criteria of Centers for Disease Control and prevention (Atlanta, GA, USA) [28]. Both ICU-acquired infections and (possible) infections upon ICU admission were evaluated for antimicrobial resistant pathogens. All admitted patients were routinely cultured on admission and sampling was repeated at 24- to 48-h intervals if there was suspected infection. Patients considered infected received antibiotic treatment empirically, pending the microbiology reports, for regularly 3–4 days. The negative culture results indicated discontinuation of antimicrobial chemotherapy; otherwise, the individual was considered infected.

ICU costs for each patient according to the national legislation were collected from the Economic Department of the Hospital (see also Additional file 1: Digital Content, Methods section, Clinical parameters; ICU infections; ICU costs).

### Evaluation of Quality-adjusted life years (QALYs)

Quality-adjusted life years (QALYs) were calculated by using the 5-level EuroQol-5-dimensional (EQ-5D-5L) questionnaire [29–31]. The study was registered at the EuroQol Research Foundation’s website (EQ-5D-5L Telephone-Paper-ID 22,972). QALY measurements were performed at 6, 12, 18 and 24 months post-ICU admission by using phone interviews; proxies were also involved in the evaluation when the patient was not able to answer [for further detail see also Additional file 1: Digital Content, Methods section, Evaluation of Quality-adjusted life years (QALYs) and Additional file 2: Fig. 1].

**Fig. 1:**
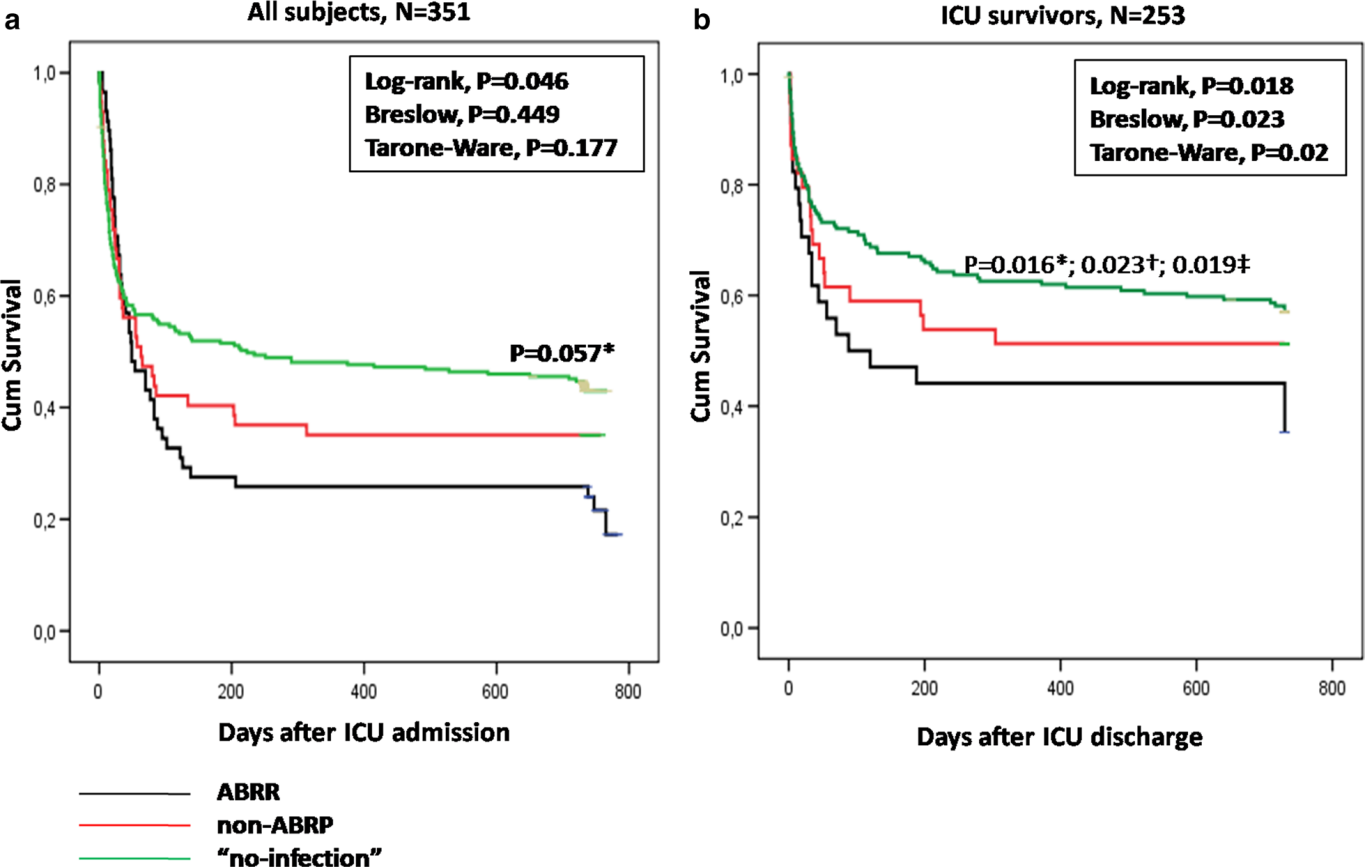
Kaplan–Meier 2-year survival curves examining the effect of ABRP infections on long-term mortality in our 351 ICU patients overall (**a**) and in the subset of 253 ICU survivors (**b**). Patients were divided into three groups according to having demonstrated ABRP infections (black line), non-ABRP infections (red line) or “no-infection” at all (green line) while in ICU. Log-rank (*), Breslow (†) and Tarone-Ware (‡) subgroup analysis between ABRP and “no-infection” group strata. *ABRP* antibiotic resistant pathogen, *ICU* intensive care unit

### Antimicrobial-resistant pathogens

Patients with ICU infections due to ABRP were included in the “ABRP group”, patients with ICU infections due to non-ABRP were included in the “non-ABRP group” whilst the ones who manifested no infection nominated the “no-infection” group [16]. Patients who were considered infected and treated empirically, but their infection was not microbiologically-confirmed (their cultures were negative), were also classified in the “no-infection” group. In case of multiple sequential infections, especially if some were caused by ABRP and some by non-ABRP, the classifying pathogen was the most resistant organism isolated over the ICU stay; thus, patients were categorized in the “ABRP group” when at least one isolate was ABRP, otherwise they were classified in the “non-ABRP” group.

ABRPs included MDR (defined as pathogens non-susceptible to at least one agent in three or more antimicrobial categories) and XDR pathogens (defined as strains non-susceptible to at least one agent in all but two or fewer antimicrobial categories) according to previously established criteria [7]. Hence, patients with multiple sequential infections due to ABRPs were further subdivided in the “XDR subgroup” when at least one isolate was XDR, otherwise they were categorized in the “MDR subgroup”.

### Study outcomes

The primary goal of our study was to examine whether antimicrobial resistance in ICU is associated with (1) increased intermediate (90-day) [22] and long-term (2-year) mortality rates, and (2) impaired quality of life for a prolonged time period (2 years post-ICU admission).

### Statistical analysis

Data is expressed as means ± standard deviation (SD), otherwise as indicated. Kolmogorov–Smirnov test was used for normality assessment. Chi-square or Fisher’s exact test were used to compare categorical variables and *t*-test or Man-Whitney *U* test to compare continuous variables as appropriate. One-way analysis of variance (ANOVA) was used for multiple comparisons between categories. Multiple regression and multivariate linear regression analyses were used to evaluate the independent clinical determinants of antimicrobial resistance, ICU costs and 2-year QALY score, as well as to exclude co-linearity among the univariate variables included in the models. To assess differences in 2-year QALY kinetics among subgroups, mean regression lines were created and compared by using linear mixed model analysis. Kaplan Meier 2-year analysis of survival was used to assess the association of ABRP, MDR/XDR pathogens and colistin administration with patients’ long-term survival. Survival curves were compared by using Log-rank test (which performs better towards the right side of the curves), Breslow test (which focuses mainly on the left side of the curves), and Tarone-Ware test (which functions better in the middle part of the curves). A Cox-proportional hazard model was constructed to ascertain whether ABRP (and XDR) infections are independent predictors of outcome or rather a function of prolonged/intensive organ support. The statistical package SPSS 17.0 (SPSS Inc., Chicago, IL, USA) was used.

## Results

A total of 373 ICU patients were assessed for eligibility to participate in the study during the study period (January 1, 2014 to December 31, 2015). Twenty-two (22) of them were excluded for several reasons (eight patients did not provide written informed consent; fourteen patients refused to participate or were not found at follow-ups, so data regarding their longitudinal course post-discharge were considered as missing); thus, 351 patients were finally enrolled and analyzed.

Fifty-eight patients (16.52%) demonstrated infection(s) due to at least one (≥ 1) ABRP (ABRP group), 57 (16.24%) manifested infection(s) due to ≥ 1 non-ABRP (non-ABRP group), while 236 (67.24%) manifested no infection at all (“no-infection” group). Eleven patients of the ABRP group (11/58; 18.9%) demonstrated microbiological results of both ABRP and non-ABRP [11]. Patients’ microbiology in our ICU population is demonstrated in detail in Table 1.

**Table 1:** Sites of ICU infections and pathogens isolated in the subsets of our study population which have demonstrated ICU infections due to either ABRP (N = 58) or non-ABRP (N = 57)

Pathogen	ICU infections				
	BSI	CRBSI	Respiratory	UTI	SSI
*ABRP group(N* = *58)*					
*In total*	57	12	2	2	2
Acinetobacter baumannii	16	7	1	–	2
Klebsiella pneumonia	32	5	–	2	–
Pseudomonas aeruginosa	6	–	1	–	–
Enterobacter cloacae	1	–	–	–	–
Staphylococcus aureus	2	–	–	–	–
*Non-ABRP group (N* = *57)*					
*In total*	59	17	8	2	–
Acinetobacter baumannii	18	3	3	–	–
Klebsiella pneumonia	9	–	4	1	–
Pseudomonas aeruginosa	7	6	–	1	–
Staphylococcus aureus	9	–	–	–	–
Staphylococcus epidermidis	10	6	–	–	–
Proteus mirabilis	1	–	–	–	–
Staphylococcus saprophyticus	2	2	–	–	–
Escherichia Coli	2	–	1	–	–
Morganella morganii	1	–	–	–	–

*ICU* intensive care unit, *ABRP* antibiotic resistant pathogen, *BSI* blood stream infection, *CRBSI* catheter related blood stream infection, *UTI* urinary tract infection, *SSI* surgical site infection

Baseline characteristics and epidemiological background of our 351 patients divided into three subgroups according to having suffered intra-ICU infections (either associated with ABRP or with non-ABRP) or not are presented in Table 2. ICU infections due to ABRP were associated significantly with more severe initial clinical condition (increased APACHE II score upon admission) but not worse preadmission status (as the latter assessed by QALY values calculated for the previous year before admission), cardiac arrest as admission etiology, as well as acute respiratory distress syndrome (ARDS), continuous renal replacement therapy (CRRT) and MODS score; among them, only MODS score (OR: 0.676, 95%CI 0.584–0.782; P < 0.001) and CRRT (OR: 4.453, 95%CI 1.805–10.982; P = 0.001) manifested independent association with the occurrence of ABRP infections in ICU (Additional file 1: Table 1). The duration of pre-infection ventilation was 11.76 ± 10.2 days in our ABRP group; sixteen out of our 19 tracheostomized patients (84.2%) developed ABRP infection before tracheostomy was performed.

**Table 2:** Epidemiological background and clinical characteristics of our study population regarding the prevalence of ICU infections [due to either ABRP (N = 58) or non-ABRP (N = 57)] or not (N = 236)

	ABRP infections N = 58	Non-ABRP infections N = 57	No infections N = 236	P-value
Age, years	66 ± 14.1	66.8 ± 13.7	65.8 ± 15.1	0.906
Sex, male (%)	43 (74.1)	38 (66.7)	145 (61.4)	0.180
*Epidemiological background*				
Previous year health-status, QALY	0.78 ± 0.13	0.77 ± 0.14	0.79 ± 0.14	0.973
Obesity	7 (12.1)	4 (7)	12(5)	0.155
COPD	10 (17.2)	12 (21.1)	50 (21.2)	0.815
Asthma	0 (0)	0 (0)	4 (1.7)	1.000
Coronary artery disease	13 (22.4)	15 (26.3)	41 (17.4)	0.265
Heart Failure	15 (25.9)	17 (29.8)	65 (27.5)	0.892
Atrial Fibrillation	8 (13.8)	4 (7)	23 (9.7)	0.472
Arterial Hypertension	14 (24.1)	16 (28.1)	69 (29.3)	0.741
Diabetes Mellitus	9(15.5)	11(19.3)	48 (20.3)	0.699
Chronic Renal Failure	2 (3.4)	3(5.3)	15 (6.3)	0.684
Previous stroke	2 (3.4)	6 (10.5)	19 (8)	0.405
Alcohol abuse	5 (8.6)	3 (5.3)	9 (3.8)	0.261
*Admitting etiology*				
Medical critical illness	37(63.8)	42(73.7)	166(70.3)	0.493
Surgical critical illness	21(36.2)	15(26.3)	70(29.7)	0.493
Cardiac arrest	7(12.1)	8 (14)	13 (5.5)	**0.038**
*Clinical characteristics*				
APACHE II on admission	21.6 ± 4.5	20.6 ± 6	18.6 ± 7.7	**0.006**^**b**^
Mechanical ventilation	51 (87.9)	50 (87.7)	184 (78)	0.085
Central line placement	54 (93.1)	53 (93)	208 (88.1)	0.364
ARDS	23 (39.7)	14 (24.6)	39 (16.5)	** < 0.001**
MODS score^27^	4.3 ± 3.7	1.7 ± 2	1.3 ± 2	** < 0.001**
Sepsis (without shock)	24 (41.4)	14 (24.6)	–	**0.047**
Septic shock	21 (36.2)	9 (15.8)	–	**0.013**
CRRT	11 (19)	7 (12.3)	14 (5.9)	**0.006**
Multiple trauma	11 (19)	6 (10.5)	25 (10.6)	0.202
Hemorrhagic Shock	3 (5.2)	3 (5.3)	15 (6.4)	0.918
Colistin administration	52 (89.7)	26 (45.6)	–	** < 0.001**
Glucocorticoid administration	19 (32.8)	12 (21.1)	59 (25)	0.333

Continuous data are presented as means ± SD, categorical data as n (%)

*ICU* intensive care unit, *ABRP* antibiotic resistant pathogen, *QALY* quality-adjusted life years, *COPD* chronic obstructive pulmonary disease, *APACHE II* Acute Physiology and Chronic Health Evaluation Score II, *ARDS* acute respiratory distress syndrome, *MODS* multiple organ dysfunction syndrome, *CRRT* continuous renal replacement therapy

^a^P < 0.05, ABRP versus non-ABRP group

^b^P < 0.05, ABRP versus no-infection group

^c^P < 0.05, non-ABRP versus no-infection group (Bonferroni’s post hoc analysis)

Intra-ICU and post-discharge outcome data in our study population overall (N = 351) as well as in the subset of ICU survivors (N = 253), divided into the three aforementioned subgroups, is presented in Table 3. ABRP infections were associated with adverse intra-ICU outcomes, including critical care myopathy and tracheostomy on discharge, prolonged mechanical ventilation and ICU stay, increased ICU costs and intra-ICU mortality (Table 3). Blood stream ABRP infections were found to constitute independent determinants of ICU cost of care (see also Additional file 1: Digital Content, Results section, as well as Additional file 1: Table 2).

**Table 3:** Intra-ICU and post-ICU outcome data in our study regarding the prevalence of ICU infections [due to either ABRP (N = 58) or non-ABRP (N = 57)] or not (N = 236)

	ABRP infections	Non-ARP infections	No infections	P-value
Population overall (N = 351)	N = 58	N = 57	N = 236	
*Intra-ICU outcomes*				
Critical Illness Myopathy	22 (37.9)	11 (19.3)	9 (3.8)	** < 0.001**
Tracheostomy on discharge	19 (15.5)	11 (19.3)	8 (3.4)	** < 0.001**
Length of stay, days	29.6 ± 21.1	16.6 ± 13.6	6.6 ± 7.7	** < 0.001**^**a,b,c**^
MV days	23.3 ± 17.6	13.1 ± 10.8	4.6 ± 6.7	** < 0.001**^**a,b,c**^
ICU cost/patient, euros	13,515 ± 7761	8330 ± 5698	4183 ± 3877	** < 0.001**^**a,b,c**^
Intra-ICU mortality	24 (41.4)	18 (31.6)	56 (23.7)	**0.022**
*Post-ICU outcomes*				
90-day mortality	42 (72.4)	33 (57.9)	109 (46.2)	**0.001**
2-year mortality	46 (79.3)	37 (64.9)	133 (56.36)	**0.005**
0.5-year QALYs	0.041 ± 0.085	0.073 ± 0.106	0.057 ± 0.097	0.077
1-year QALYs	0.105 ± 0.219	0.180 ± 0.265	0.142 ± 0.245	0.098
1.5-year QALYs	0.167 ± 0.358	0.289 ± 0.433	0.228 ± 0.4	0.101
2-year QALYs	0.228 ± 0.501	0.399 ± 0.604	0.313 ± 0.559	0.101
*ICU survivors (N* = *253)*	N = 34	N = 39	N = 180	
90-day mortality	18 (52.9)	15 (38.5)	53 (29.4)	**0.024**
2-year mortality	22 (64.7)	19 (48.7)	103 (57.2)	0.061
0.5-year QALYs	0.070 ± 0.102	0.107 ± 0.113	0.159 ± 0.142	**0.001**^**b**^
1-year QALYs	0.179 ± 0.263	0.264 ± 0.285	0.380 ± 0.341	**0.002 **^**b**^
1.5-year QALYs	0.285 ± 0.432	0.423 ± 0.468	0.594 ± 0.543	**0.003 **^**b**^
2-year QALYs	0.389 ± 0.608	0.584 ± 0.654	0.808 ± 0.747	**0.004 **^**b**^

Continuous data are presented as means ± SD, categorical data as n (%)

*ICU* intensive care unit, *ABRP* antibiotic resistant pathogen, *MV days* days on mechanical ventilation, *QALY* quality-adjusted life years

^a^P < 0.05, ABRP versus non-ABRP group

^b^P < 0.05, ABRP versus no-infection group

^c^P < 0.05, non-ABRP versus no-infection group (Bonferroni’s post hoc analysis)

The ABRP group was further divided into MDR subgroup (N = 34) and XDR subgroup (N = 24; 8 of them manifested both XDR/MDR isolates). XDR subgroup was associated with more days on mechanical ventilation (31.9 ± 21 vs. 17.3 ± 11.5, P = 0.001), prolonged ICU length of stay (39.5 ± 24.5 vs. 22.6 ± 15.1, P = 0.002), higher intra-ICU mortality [62.5% (15/24) vs. 26.5% (9/34), P = 0.006] and higher MODS score (5.7 ± 3.9 vs. 3.3 ± 3.3, P = 0.016), yet similar APACHE II on admission (22.1 ± 4.9 vs. 21.2 ± 4.1, P = 0.431) compared to MDR subset.

Interestingly, patients who developed ICU infections due to XDR pathogens were of similar age (63.3 ± 13.8 vs. 66.2 ± 14.7, P = 0.355), predominantly males [22/24 (91.7%) vs. 209/327 (63.9%), P = 0.006], and demonstrated higher APACHE II on admission (22.1 ± 4.9 vs. 19.2 ± 7.2, P = 0.05) and MODS score (5.7 ± 3.9 vs. 1.6 ± 2.2, P < 0.001), compared to the rest ICU population.

### Association between antimicrobial resistance and long-term survival and quality of life

In our study population, intra-ICU, 90-day and 2-year overall mortality were 27.9% (98/351), 52.4% (184/351) and 61.5% (216/351), respectively. ABRP infections not only were associated with increased ICU mortality, but also with enhanced 90-day and 2-year mortality rates (Table 3). XDR subgroup was associated with increased 90-day and 2-year mortality compared to MDR subset (Additional file 3: Fig. 2).

**Fig. 2:**
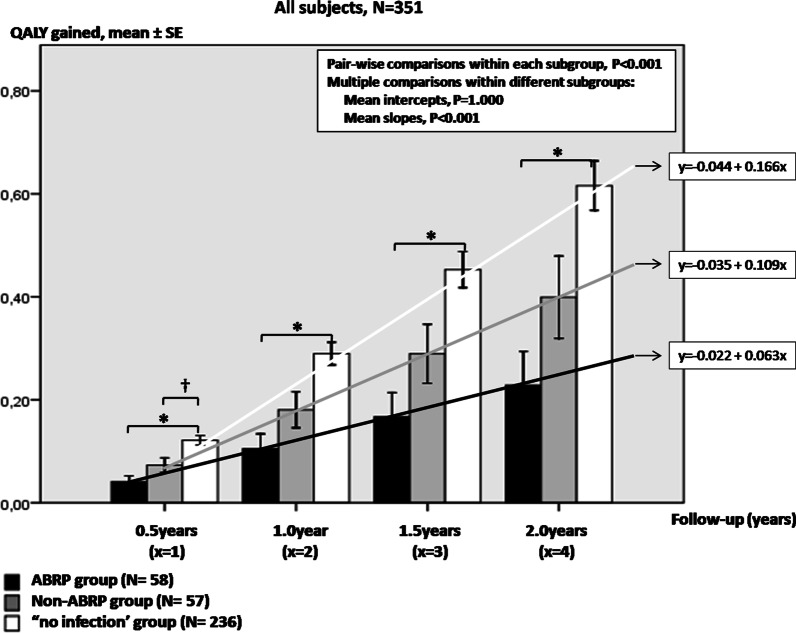
Two-year QALY kinetics in ABRP, non-ABRP and “no-infection” groups in our 351 patients overall. Bars and vertical lines indicate mean QALY values and standard errors, respectively. QALY kinetics is indicated by the corresponding mean regression line for ABRP group (black line), non-ABRP group (gray line) and “no-infection” group (white line). The ABRP group displays the worse progress in QALY kinetics over time (lower slope of its corresponding mean regression line) compared to the two other groups. QALY, quality-adjusted life years; SE, standard error; ABRP, antibiotic resistant pathogen; Intercept of the regression line, the QALY value where the regression line crosses the y-axis at the theoretical day, 0; Slope of the regression line, the rate at which QALY values change between two-consequent follow-up examinations. *P < 0.001; †P < 0.05

Figure 1 illustrates the Kaplan–Meier 2-year survival analysis in our 351 ICU patients overall, as well as in the subset of 253 ICU survivors, respectively, divided into the three abovementioned (ABRP, non-ABRP and “no-infection”) subgroups. ABRP infections were associated with increased rates of delayed mortality in ICU patients overall (Log-rank test, P = 0.046) (panel A). Interestingly, when analysis was restricted only in the subset of ICU survivors, ABRP infections were markedly associated with short-term, intermediate and long-term mortality post-discharge [as assessed by Breslow (P = 0.023) Tarone-Ware (P = 0.02), and Log-rank (P = 0.018) tests, respectively] (panel B). Notably (see Additional file 4: Fig. 3), the longitudinal adverse effect of antimicrobial resistance was mainly due to XDR rather than MDR pathogens, especially in the subset of ICU survivors (Log-rank, P = 0.018). Remarkably, Cox-proportional hazard analyses demonstrate that ABRP infections overall (Table 4; Model 1), as well as XDR (Table 4; Model 2) but not MDR (Additional file 1: Table 3) infections, may represent independent predictors of long-term mortality, after adjusting for some indicators of the severity of underlying critical illness.

**Table 4:** Cox regression survival analysis examining the effect of ABRP and XDR infections (Model 1 and 2, respectively) on 2-year mortality, after adjusting for possible covariates of severe underlying illness

	Model 1				Model 2			
	HR	95% CI		P value	HR	95% CI		P value
		Lower	Upper			Lower	Upper	
APACHE II	1.099	1.075	1.124	** < 0.001**	1.100	1.076	1.125	** < 0.001**
MODS score^27^	1.146	1.083	1.213	** < 0.001**	1.140	1.077	1.207	** < 0.001**
ARDS	0.786	0.553	1.115	0.177	0.797	0.563	1.128	0.200
CRRT	1.248	0.776	2.007	0.360	1.384	0.866	2.213	0.174
MV prior to infection	0.741	0.494	1.111	0.147	0.707	0.470	1.062	0.095
Cardiac arrest	1.030	0.641	1.657	0.901	1.026	0.638	1.650	0.915
ABRP infections	1.778	1.166	2.711	**0.008**	–	–	–	–
XDR infections	–	–	–	–	1.889	1.075	3.320	**0.027**

*ABRP* antibiotic resistant pathogens, *XDR* extensively-drug resistant pathogens, *CI* confidence interval, *HR* hazard ratio, *APACHE II* Acute Physiology and Chronic Health Evaluation Score II, *MODS* multiple organ dysfunction syndrome, *ARDS* acute respiratory distress syndrome, *CRRT* continuous renal replacement therapy, *MV* mechanical ventilation

Variables included in the Models were assessed for multicolinearity issues; All Variable Inflation Factors (VIF) ≤ 1.796

ICU infection(s) due to ABRP(s) were associated with worse QALY kinetics not only in our ICU population overall (Fig. 2), but also when analysis was restricted in the subset of ICU survivors (Additional file 5: Fig. 4) or even in the one of 2-year survivors (Additional file 6: Fig. 5). Interestingly, XDR and MDR subgroups demonstrated no differences in QALY values either in 90-day or in 2-year survivors (Additional file 3: Fig. 2). Multivariate regression analysis in Additional file 1: Table 4 indicates that antimicrobial resistance is not an independent determinant of long-term quality of life but rather a function of the severity of critical illness.

Patients’ ABRP specific microbiology showed no association with either mortality or QALY values (Additional file 7: Fig. 6).

Colistin administration was associated with increased longitudinal mortality rates (Additional file 8: Fig. 7) in ICU patients overall (log-rank, P = 0.049), but mainly in patients having survived ICU (Log-rank, P = 0.018). In our series, 78 patients overall required colistin treatment: 23 (29.5%) due to XDR and 55 (70.5%) due to MDR/nonABRP infections; a Kaplan–Meier 2-year survival analysis between the two subgroups (Additional file 9: Fig. 8) showed that XDR pathogens are likely to be associated with worse outcomes compared to other (MDR/non-ABRP) pathogens also requiring colistin administration (Log-rank, P = 0.021). Colistin was also found to influence independently ICU cost of care (see also Additional file 1: Digital Content, Results section, ICU cost of care, as well as Additional file 1: Table 2).

## Discussion

The most important finding of this study is that ICU infections due to antimicrobial resistant pathogens may exert a prolonged adverse impact on critically ill patients’ longevity and well-being post-ICU discharge. This information is important, since it may indicate that despite the implementation of intensive care treatment, patients who develop infections due to ABRPs while in ICU are expected to have limited benefits in patient-important quality-of-life outcomes and long-term survival. Interestingly, our findings may also suggest that XDR infections represent an independent determinant of long-term survival rather than a mediating factor in declining health status of patients with more severe critical illness; however, this remains to be elucidated in larger-scale future studies.

The present study provides evidence that antimicrobial resistance in ICU is associated with adverse intra-ICU outcomes, including prolonged mechanical ventilation and ICU length of stay, and increased ICU costs [32]; in addition, our results may suggest that ABRP infections possibly impact negatively ICU mortality (Table 3). These data is in line with previous reports [10–14], while no significant impact on ICU mortality was found in other studies [9, 15–19]. Remarkably, a number of these studies were Greek as well [9, 10, 14, 18, 19], and mainly included gram-negative ABRPs [9–19]. However, there is still limited data in the literature regarding the relationship between ABRP infections and mortality rates post-discharge [33].

In our series, intra-ICU mortality was 27.9%, and rose abruptly to 52.4% in only 90 days post-ICU admission. Beyond 90-days, long term-mortality reached a “plateau” phase, rising at a value of 61.5% at 2-year follow-up. Our results are in line with previous reports, also suggesting unacceptably high mortality rates post-ICU discharge [34]. This has been attributed to multiple reasons, such as higher pathogen virulence and/or weaker hosts [35, 36], specific type of infections (such as pneumonia or peritonitis) [37], inappropriate empirical antimicrobial therapy and/or delays in administering effective antimicrobial treatment [19, 38, 39]. Moreover, inability of the staff in lower levels of care to keep up with the needs of critically ill patients [40], absence of Intermediate Care Unit facilities [34] and premature discharge from ICU due to the constant need for beds [41] may be related to enhanced mortality rates post-discharge. However, the longitudinal impact of antimicrobial resistance on both intermediate and long term survival has not been examined so far. Our series indicates that ABRP infections in ICU (especially due to XDRs) may be associated with increased 90-day and 2-year mortality (Table 3 and Additional file 3: Fig. 2). In addition, ABRP group (mainly XDR subgroup) demonstrated worse survival curves not only in ICU patients overall, but also in the subset of ICU survivors; in the latter, it is of note that antimicrobial (possibly XDR) resistance was associated with increased short-term, intermediate as well as long-term mortality post-discharge (Fig. 1 and Additional file 4: Fig. 3). Accordingly, one could argue that ICU survivors with a history remarkable for antimicrobial resistance while in ICU may continuously be at high risk for restricted life expectancy post-discharge; however, this hypothesis, as well as the possible mechanisms mediating this relationship, warrant further investigation in future studies.

In the recent years, patient-important outcomes are gaining wide acceptance in most fields of clinical research. Apart from decreasing mortality, clinical decision-making also aims at improving quality of life and functional status in ICU survivors [4, 23]. Up to the present, several aspects of critical illness have been investigated as possible determinants of long term outcomes (including QALY assessment) post-ICU discharge, such as the duration of mechanical ventilation/ICU stay [42, 43], septic shock [44, 45], ARDS [46], COPD [47], cardiac arrest on admission [48] etc. To the best of our knowledge, the possible role of antimicrobial resistance on quality-of-life, physical and psychological well-being for a prolonged period post-ICU discharge is examined for the first time in the present investigation. Interestingly, our study may suggest that antimicrobial resistance is likely to be associated with significant impairment in health-related quality-of-life for months, even years after ICU admission. Remarkably, ABRP infections in our series were associated with reduced 6-month to 2-year QALY kinetics (Fig. 2, Additional files 5 and 6: Figs. 4 and 5); similarly to long-term mortality, this relationship mainly concerned patients who managed to survive ICU (Table 3, Additional file 5: Fig. 4); in this respect, our findings are important, since it is suggested that history of ABRP infections in ICU, apart from increased mortality, may also predict limited anticipated benefits in non-neurocritical (as was the case in our study) critically ill patients post-discharge.

Antimicrobial resistance was associated with several aspects of critical illness (Table 2) and prolonged mechanical ventilation/ICU stay in our series (Table 3). Certainly, ABRP infections occurred predominantly in the early phase of critical illness rather than in the late stage of ICU stay (as indicated by relatively short duration of pre-infection ventilation and higher incidence of antimicrobial resistance before tracheostomy). Undoubtedly, the more severe critical illness may have predisposed to both ABRP infections and adverse long-term outcomes [33]; on the other hand, ABRP infections may have resulted in a more complicated and prolonged clinical course, which in turn may have led to severe persistent impairments and functional limitations. Interestingly, our study provides evidence that even in “sicker” ICU patients, infections due to either ABRPs or XDR pathogens may bear additional risk for increased long term (2-year) mortality (Table 4), but not affect 2-year QALY scores independently (Additional file 1: Table 4). Notably, the harmful effect of antimicrobial resistance (after adjusting for possible covariates of severe underlying illness) on long-term mortality is likely to be restricted to the XDR organisms rather than the MDR subset (Table 4, Additional file 1: Table 3, and Additional file 4: Fig. 3). Furthermore, specific therapeutic approaches aiming at treating resistant pathogens, such as colistin administration (Additional file 8: Fig. 7), may have also influenced long-term outcomes. Of course, colistin was the main regimen used for ABRP infections, hence its possible long-term consequences possibly reflect the ones of coinciding antimicrobial resistance (especially of XDR organisms, Additional file 9: Fig. 8). Finally, post-ICU care facilities and rehabilitation centers may contain numerous niches of MDR/XDR bacteria (i.e. infected patients, biofilms, toilets, surfaces, keyboards), which in turn may constitute “virulence-like” community-resistance reservoirs for secondary infection outbreaks [8, 44]. Nevertheless, our limited data may indicate a negative effect of antimicrobial resistance in declining health status, especially in the subset of the more severe critically ill. In this respect, prevention against ABRP infections might be of fundamental importance in modifying the clinical course of patients with an “a priori” dismal prognosis.

A few limitations to the study deserve mention. First, we used consecutive two-year sampling in order to have valid and meaningful results on the role of antimicrobial resistance in affecting long-term outcomes; however, we certainly admit that our findings (especially on the role of XDRs on critically ill patients’ longevity post-discharge) should be validated in larger-scale studies in the future. Second, most of our infections were non-catheter-related blood stream infections, while respiratory infections were far less common in our cohort. One could argue that the synthesis of our ICU infections and its specific microbiology (mainly gram negative bacteria) may have interfered with our findings. Certainly, genetic and geographic differences may exert diverse influence on the clinical phenotypes of special diseases in different populations. Furthermore, it has been shown [49] that some organ failures (i.e. cardiovascular system) may adversely affect outcomes of ICU patients more than others; however, the MODS score we used was inadequate to examine potential longitudinal differences among specific organ failures. Moreover, a drawback of the study is the likelihood of misclassifying a culture-negative infection as no infection at all. However, this possibility was small, by carefully culturing in concert with the routine microbiological sampling of the department. Finally, meningitis/ventriculitis due to ABRPs, which has shown significant diagnostic performance in post-neurosurgical patients [50], was not examined in our series.

## Conclusions

The present prospective study demonstrated a significant association between ABRP (especially XDR) infections in ICU and increased mortality/inability rates for a prolonged period post-discharge. This relationship is likely to mainly concern patients with more severe critical illness, who manage to survive ICU though; in this respect, a history remarkable of antimicrobial resistance while in ICU should raise awareness for increased risk post-discharge and reconsider the anticipated benefits in long-term longevity and well-being, especially in patients with more severe critical illness and/or complex course of hospitalization.

## Supplementary Information

**Additional file 1**. Details in methodology used, results obtained.

**Additional file 2**: **Supplemental Figure 1** Examples of calculation of QALY-gained in our study. A. QALYs-gained by ICU treatment in a patient having survived the 2-year follow-up (QALYs gained= area A + area B + area C + area D). B. QALYs-gained by ICU treatment in the theoretical patient who died between the 12-month and the 18-month follow-up (QALYs gained= area A+ area B + area C). HRQoL= health-related quality of life; HRQoL_1,2,3,4_= utility index values at 6,12,18 and 24-month follow-up, respectively; ICU= intensive care unit; QALYs= quality-adjusted life years.

**Additional file 3**: **Supplemental Figure 2**. Long-term outcomes in the MDR/XDR subgroups of our 58 patients with antibiotic resistant pathogens. In the upper panel, bars represent the number of 90-day (A) and 2-year (B) survivors and non-survivors with MDR and XDR infections; percentages within grey bars represent mortality in each category. In the lower panel, bars and vertical lines indicate mean QALY values and standard errors in 90-day and 2-year survivors, respectively. MDR= multi-drug resistant pathogen; XDR= extensively-drug resistant pathogen; QALY= quality-adjusted life years.

**Additional file 4**: **Supplemental Figure 3**. Kaplan-Meier 2-year survival curves examining the effect of ABRP infections on long-term mortality post-discharge. Analysis was performed in our 351 ICU patients overall (panel A), and in the subset of 253 ICU survivors (panel B). Patients were divided into four groups according to having demonstrated MDR infection(s) (black dotted line), XDR infection(s) (continuous black line), infections due to non-ABRPs (red line) or “no-infection” at all (green line) while in ICU. ABRP= antibiotic resistant pathogen; MDR= multi-drug resistant; XDR= extensively-drug resistant; ICU= intensive care unit.

**Additional file 5**: **Supplemental Figure 4**. Two-year QALY kinetics in ABRP, non-ABRP and “no-infection” subgroups of our ICU survivors (N=253). Bars and vertical lines indicate mean QALY values and standard errors, respectively. ABRP group demonstrates lower increase in QALYs over time (markedly depressed slope of the corresponding mean regression line) compared to its counterparts. QALY= quality-adjusted life years; ABRP= antibiotic resistant pathogen; ICU= intensive care unit; SE= standard error; Intercept of the regression line= the QALY value where the regression line crosses the y-axis at the theoretical day=0; Slope of the regression line= the rate at which QALY values change between two-consequent follow-up examinations. *P<0.001.

**Additional file 6**: **Supplemental Figure 5**. Two-year QALY kinetics in ABRP, non-ABRP and “no-infection” subgroups of our 2-year survivors (N=135). Bars and vertical lines indicate mean QALY values and standard errors, respectively. ABRP group demonstrates lower increase in QALYs over time (lower slope of the corresponding mean regression line) compared to its counterparts. QALY= quality-adjusted life years; ABRP= antibiotic resistant pathogen; SE= standard error; Intercept of the regression line= the QALY value where the regression line crosses the y-axis at the theoretical day=0; Slope of the regression line= the rate at which QALY values change between two-consequent follow-up examinations.*P<0.05.

**Additional file 7**: **Supplemental Figure 6**. Long-term outcomes in our 58 ABRP patients according to underlying pathogen. In the upper panel, bars represent number of 90-day (A) and 2-year (B) survivors and non-survivors with respect to underlying pathogen(s). In the lower panel, bars and vertical lines indicate mean QALY values and standard errors in 90-day and 2-year survivors regarding the underlying pathogen(s), respectively. ABRP= antibiotic resistant pathogens; QALY= quality-adjusted life years; Ab=Acinetobacter baumannii; Ent/ter=Enterobacter cloacae; KPC= Klebsiella pneumonia producing carbapenemases; Pa= Pseudomonas aeruginosa; Sa= staphylococcus aureus.

**Additional file 8**: **Supplemental Figure 7**. Kaplan-Meier survival curves examining the effect of colistin administration in ICU on long-term mortality post-discharge. Analysis was performed in our 351 ICU patients overall (panel A), and in the subset of 253 ICU survivors (panel B). ICU= intensive care unit.

**Additional file 9**: **Supplemental Figure 8**. Kaplan-Meier survival curves examining the effect of XDR versus non-XDR (MDR plus non-ABRP) infections on 2-year mortality in our 78 patients treated with colistin. XDR= extensively-drug resistant pathogens; MDR= multidrug resistant pathogens; ABRP= antibiotic resistant pathogens.

## Data Availability

The datasets used and/or analysed during the current study are available from the corresponding author on reasonable request.

## Abbreviations

ICU: Intensive Care Unit
ABRP: Antibiotic resistant pathogen
HR: Hazard ratio
MDR: Multi-drug resistant
XDR: Extensively-drug resistant
QALY: Quality-adjusted life year
OR: Odds ratio
CI: Confidence interval
APACHE II: Acute Physiology and Chronic Health Evaluation II
HRQoL: Health related quality of life
EQ-5D-5L: EuroQol-5 dimensions-5 levels
SD: Standard deviation
MODS: Multiple organ dysfunction syndrome
LOS: Length of ICU stay
