# Supplementary material for: The role of antimicrobial resistance on long-term mortality and quality of life in critically ill patients: a prospective longitudinal 2-year study
Source: Health Qual Life Outcomes. 2021 Mar 3;19:72. doi: 10.1186/s12955-021-01712-0 (PMC7927260; doi:10.1186/s12955-021-01712-0)
Supplement: Supplementary file 1 — Additional file 1. Details in methodology used, results obtained. [file 12955_2021_1712_MOESM1_ESM.docx]

**Supplemental Digital Content**

**The role of antimicrobial resistance on long-term mortality and quality of life in critically ill patients: a prospective longitudinal 2-year study**

Triantafyllia Koukoubani^1^, Demosthenes Makris^2^, Zoe Daniil^2^, Theoniki Paraforou^1^, Vasiliki Tsolaki^2^, Epaminondas Zakynthinos^2^, John Papanikolaou^2^

^1^ Department of Critical Care, General Hospital of Trikala, Thessaly, Greece

^2^ Department of Critical Care, School of Medicine, University of Thessaly, University Hospital of Larissa, Thessaly, Greece

**Methods**

***Clinical parameters***

Acute Physiology and Chronic Health Evaluation (APACHE II score) was calculated on admission in ICU [*E1*].

Cardiac arrest [*E2*], hemorrhagic shock [*E3*] and ARDS [*E4*] were recorded.

Comorbidities including diabetes, previous stroke, hypertension, coronary artery disease, atrial fibrillation, valvular heart disease, heart failure, chronic obstructive pulmonary disease (COPD), asthma, chronic renal failure etc. were also recorded. Obesity was present if Body Mass Index (BMI)≥30kg/m^2^ was found [*E5*].

Acute renal failure was identified if RIFLE (Risk, Injury, Failure, Loss of kidney function, and End-stage kidney disease) criteria for acute kidney injury were present [*E6*]. Critical illness myopathy (CIM) [*E7*] was identified according to the published criteria.

Mechanical ventilation days were defined as the period from the first day of intubation and mechanical ventilation until the extubation day [*E8*].Tracheostomy was performed in selected patients who either had difficulty/multiple failures in weaning from mechanical ventilation or presented impaired neurological function.

The terms sepsis and septic shock were utilized and the records were revised according to existing literature [*E9*]. Glucocorticoids were given according to the existing guidelines [*E10*]. Colistin was recorded if given either based on microbiology results or empirically.

***Evaluation of Quality-adjusted life years (QALYs)***

1. ***The EQ-5D-5L instrument***

The 5- level EuroQol 5-dimensional questionnaire (EQ-5D-5L) was introduced by the EuroQol Group in 2009 to improve the instrument’s sensitivity and to reduce the “ceiling” effect (state of perfect health). Five dimensions are included: mobility, self-care, usual activities, pain or discomfort, and anxiety or depression. Each dimension is divided in 5 levels: no problems, slight problems, moderate problems, severe problems and extreme problems [*E11*]. The digits for the five dimensions form a 5-digit number that describes the patient’s health state quite analytically [*E11*].

1. ***Calculation of QALYs***

The calculation of QALYs is based on quality of life scores (“utilities”) generated from health-related quality of life (HRQoL) questionnaires at baseline and at specific time points during the follow-up. In our study, “utilities” were estimated using the 5-level EuroQol-5-dimensional (EQ-5D-5L) questionnaire, and expressed as numbers ranging from -0.53 to 1, where zero applies to the state of death, one reflects the state of perfect health, whilst negative scores indicate health states worse than death [*E11-E13*]. The assessment of QALY score for each patient in our study was based on the second assumption used for calculation of QALYs by Vainiola and co-workers [*E14*]. In brief, a critically ill patient without admission/treatment in ICU would die immediately, gaining a QALY value of zero. Instead, ICU treatment would result in a linear augmentation of HRQoL score from baseline to the first follow-up and subsequently between consecutive follow-upsaccording to EQ-5D-5L Index Value Calculator developed by the EuroQol Group [*E15*]. Thus, a patient who has died before the 6-month first follow-up scores a QALY value of zero. Instead, patients having survived the 6-month first follow-up manage to gain certain QALY values. According to the mentioned methodology [*E14*], the QALYs gained at a specific time point are calculated from the shaded area (*Supplemental Figure 1*) under the formed curve and left to this time point (HRQoL score vs. time graph). Thus, a patient who survives the final 2-year follow-up of our study (*Supplemental Figure 1*, panel A) gains a QALY value that equals the sum of area A (triangle) + area B (trapezium) + area C (trapezium) + area D (trapezium). Instead, a patient who has died between the 12-month and 18-month follow-up (*Supplemental Figure 1*, panel B) gets a QALY value which equals the sum of area A (triangle) + area B (trapezium) + area C (triangle) [*E14*]. Theoretically, the maximum number of QALYs gained is 1.875 [(1*0.5)/2 + 1*1.5= 1.75)].

***ICU infections***

Infections were defined according to criteria of Centers for Disease Control and prevention (Atlanta, GA, USA) [*E16*]. Quantitative cultures, microbiologic blood examination, as well as cytology, microbiology and biochemistry of drained fluids were performed when the patient was considered to be infected at physicians’ discretion.

Clinically significant blood stream infections (BSI) was deemed to be present if a pathogenic organism was cultured from at least one set of blood cultures [*E17*]. The diagnosis of BSI due to common skin contaminants (Coagulase Negative Staphylococci, Bacillus, Corynebacterium or Propionibacterium species) required at least two positive sets of blood cultures and one of the signs-symptoms of sepsis (fever>38°C, chills or rigors and hypotension) within 24 hours of a positive blood culture [*E16*].

The diagnosis of catheter related BSI (CRBSI) requires a positive culture of blood from a peripheral vein and clear evidence that the catheter is the source. CRBSI means a patient with an intravascular catheter has at least one positive blood culture obtained from a peripheral vein, clinical manifestations of infection (i.e., fever, chills, and/or hypotension), and no apparent source for the BSI, except the catheter. In addition, a positive semi-quantitative [>15 colony-forming units (CFU) /catheter segment][*E18*] or quantitative (>10^3^CFU/catheter segment)[*E19*] catheter tip culture may be also present, provided that the same organism (species and anti-biogram) is isolated from the catheter segment and peripheral blood culture.

Respiratory infections included ventilator-associated pneumonia and ventilator-associated tracheobronchitis [*E16*]. Ventilator-associated pneumonia was defined as the presence of new or progressive radiographic infiltrate in combination with two of the three following criteria: (1) temperature >38^o^C (2) white blood cell (WBC) count>12000/mm^3^ or <4000/ mm^3^, and (3) purulent tracheal aspirate. In addition, a positive tracheal aspirate in quantitative cultures (≥10^5^CFU/mL) or a positive bronchoalveolar lavage culture (≥10^4^ CFU/mL) was required to confirm the diagnosis [*E16*]. Ventilator-associated tracheobronchitis was defined as fever (>38°C) with no other recognizable cause, purulent sputum production, positive endotracheal aspirate culture (≥10^5^CFU/mL), but no radiographic signs of new pneumonia [*E20*].

Surgical site infection (SSI) was diagnosed if a) organisms had been cultured from tissue or drainage from affected site, b) purulent drainage was obtained from affected site, and c) patients had at least two of the following signs or symptoms at the affected site with no other recognized cause: localized pain or tenderness, redness, swelling, or heat [*E16*].

Urinary tract infection (UTI) was defined as sepsis with fever(>38°C), pyuria (urine specimen with ≥10 WBC/mm^3^ of unspun urine or >5 WBC/high power field of spun urine) and a positive culture ≥10^5^CFU/mL [*E16*], with no other obvious site of infection.

***ICU costs***

Costs were obtained according to the study period (2014-2015) available tariffs of diagnostic tests, certain therapeutic schemes and fixed reimbursement rate (direct medical costs only) for critically ill patients in Greek ICUs. Costs generating from emergency department, operating and recovery rooms as well as the fixed ICU costs (i.e. operational costs, staff salaries) were excluded [*E21-E22*]. ICU costs were collected from the Economic Department of the Hospital after appropriate authorization.

**Results**

***ICU cost of care***

ICU infections due to antibiotic-resistant pathogens (ABRP) were associated with ICU costs [13515±1019 vs. 8330±755, vs.4182±252€, for ABRP, non-ABRP and “no-infection” groups, respectively, P_ANOVA_<0.001 (P<0.001 for ABRP vs. the other two groups, Bonferoni post-hoc analysis) (Table 3, main text). Among several clinical factors significantly associated with ICU cost of care on univariate analysis, ABRP-associated blood stream infections, along with cardiac arrest, tracheostomy, critical illness myopathy and colistin administration were independent determinants of ICU cost on multivariate linear regression analysis (*Supplemental Table 2*).

***References (Supplemental Digital Content)***

1. Marsh HM, Krishan I, Naessens JM [Strickland RA](https://www.ncbi.nlm.nih.gov/pubmed/?term=Strickland%20RA%5BAuthor%5D&cauthor=true&cauthor_uid=2123955), [Gracey DR](https://www.ncbi.nlm.nih.gov/pubmed/?term=Gracey%20DR%5BAuthor%5D&cauthor=true&cauthor_uid=2123955), [Campion ME](https://www.ncbi.nlm.nih.gov/pubmed/?term=Campion%20ME%5BAuthor%5D&cauthor=true&cauthor_uid=2123955), [Nobrega FT](https://www.ncbi.nlm.nih.gov/pubmed/?term=Nobrega%20FT%5BAuthor%5D&cauthor=true&cauthor_uid=2123955), [Southorn PA](https://www.ncbi.nlm.nih.gov/pubmed/?term=Southorn%20PA%5BAuthor%5D&cauthor=true&cauthor_uid=2123955), [McMichan JC](https://www.ncbi.nlm.nih.gov/pubmed/?term=McMichan%20JC%5BAuthor%5D&cauthor=true&cauthor_uid=2123955), [Kelly MP](https://www.ncbi.nlm.nih.gov/pubmed/?term=Kelly%20MP%5BAuthor%5D&cauthor=true&cauthor_uid=2123955) (1990) Assessment of prediction of mortality by using the APACHE II scoring system in intensive-care units. *Mayo ClinProc.* 65:1549-1557.
2. Panchal A, Berg K, Kudenchuk P, Del Rios M, Hirsch K, Link M, Kurz M, Chan P, Cabanas J, Morley P, Hazinski M, Donnino M (2018) 2018 American Heart Association Focused Update on Advanced Cardiovascular Life Support Use of Antiarrhythmic Drugs During and Immediately After Cardiac Arrest: An Update to the American Heart Association Guidelines for Cardiopulmonary Resuscitation and Emergency Cardiovascular Care.*Circulation.* 138:e740-e749.
3. Cannon JW (2018) Hemorrhagic Shock. *N Engl J Med*. 378:370-379.
4. FanelliV, Vlachou A, Ghannadian S, Simonetti U, Slutsky AS, Zhang H (2013;) Acute respiratory distress syndrome: new definition, current and future therapeutic options. *J Thorac Dis*. 5(3):326-334.
5. [Pi-Sunyer FX](https://www.ncbi.nlm.nih.gov/pubmed/?term=Pi-Sunyer%20FX%5BAuthor%5D&cauthor=true&cauthor_uid=11115784) (2000) Obesity: criteria and classification. [*ProcNutr Soc.*](https://www.ncbi.nlm.nih.gov/pubmed/?term=pi-sunyer+PROCEEDINGS+OF+THE+NUTRITION+SOCIETY+2000+59+505-509)  59(4):505-509.
6. [Hoste EA](https://www.ncbi.nlm.nih.gov/pubmed/?term=Hoste%20EA%5BAuthor%5D&cauthor=true&cauthor_uid=16696865), [Clermont G](https://www.ncbi.nlm.nih.gov/pubmed/?term=Clermont%20G%5BAuthor%5D&cauthor=true&cauthor_uid=16696865), [Kersten A](https://www.ncbi.nlm.nih.gov/pubmed/?term=Kersten%20A%5BAuthor%5D&cauthor=true&cauthor_uid=16696865), [Venkataraman R](https://www.ncbi.nlm.nih.gov/pubmed/?term=Venkataraman%20R%5BAuthor%5D&cauthor=true&cauthor_uid=16696865), [Angus DC](https://www.ncbi.nlm.nih.gov/pubmed/?term=Angus%20DC%5BAuthor%5D&cauthor=true&cauthor_uid=16696865), [De Bacquer D](https://www.ncbi.nlm.nih.gov/pubmed/?term=De%20Bacquer%20D%5BAuthor%5D&cauthor=true&cauthor_uid=16696865), [Kellum JA](https://www.ncbi.nlm.nih.gov/pubmed/?term=Kellum%20JA%5BAuthor%5D&cauthor=true&cauthor_uid=16696865) (2006) RIFLE criteria for acute kidney injury are associated with hospital mortality in critically ill patients: a cohort analysis. [*Crit Care.*](https://www.ncbi.nlm.nih.gov/pubmed/16696865)  10(3):R73.
7. [Shepherd S](https://www.ncbi.nlm.nih.gov/pubmed/?term=Shepherd%20S%5BAuthor%5D&cauthor=true&cauthor_uid=28042370), [Batra A](https://www.ncbi.nlm.nih.gov/pubmed/?term=Batra%20A%5BAuthor%5D&cauthor=true&cauthor_uid=28042370), [Lerner DP](https://www.ncbi.nlm.nih.gov/pubmed/?term=Lerner%20DP%5BAuthor%5D&cauthor=true&cauthor_uid=28042370) (2017) Review of critical illness myopathy and neuropathy. [*Neurohospitalist.*](https://www.ncbi.nlm.nih.gov/pubmed/28042370)  7(1):41-48.
8. [Rose L](https://www.ncbi.nlm.nih.gov/pubmed/?term=Rose%20L%5BAuthor%5D&cauthor=true&cauthor_uid=28611229), [McGinlay M](https://www.ncbi.nlm.nih.gov/pubmed/?term=McGinlay%20M%5BAuthor%5D&cauthor=true&cauthor_uid=28611229), [Amin R](https://www.ncbi.nlm.nih.gov/pubmed/?term=Amin%20R%5BAuthor%5D&cauthor=true&cauthor_uid=28611229), [Burns KE](https://www.ncbi.nlm.nih.gov/pubmed/?term=Burns%20KE%5BAuthor%5D&cauthor=true&cauthor_uid=28611229), [Connolly B](https://www.ncbi.nlm.nih.gov/pubmed/?term=Connolly%20B%5BAuthor%5D&cauthor=true&cauthor_uid=28611229), [Hart N](https://www.ncbi.nlm.nih.gov/pubmed/?term=Hart%20N%5BAuthor%5D&cauthor=true&cauthor_uid=28611229), [Jouvet P](https://www.ncbi.nlm.nih.gov/pubmed/?term=Jouvet%20P%5BAuthor%5D&cauthor=true&cauthor_uid=28611229), [Katz S](https://www.ncbi.nlm.nih.gov/pubmed/?term=Katz%20S%5BAuthor%5D&cauthor=true&cauthor_uid=28611229), [Leasa D](https://www.ncbi.nlm.nih.gov/pubmed/?term=Leasa%20D%5BAuthor%5D&cauthor=true&cauthor_uid=28611229), [Mawdsley C](https://www.ncbi.nlm.nih.gov/pubmed/?term=Mawdsley%20C%5BAuthor%5D&cauthor=true&cauthor_uid=28611229), [McAuley DF](https://www.ncbi.nlm.nih.gov/pubmed/?term=McAuley%20DF%5BAuthor%5D&cauthor=true&cauthor_uid=28611229), [Schultz MJ](https://www.ncbi.nlm.nih.gov/pubmed/?term=Schultz%20MJ%5BAuthor%5D&cauthor=true&cauthor_uid=28611229), [Blackwood B](https://www.ncbi.nlm.nih.gov/pubmed/?term=Blackwood%20B%5BAuthor%5D&cauthor=true&cauthor_uid=28611229) (2017) Variation in definition of prolonged mechanical ventilation. [*Respir Care.*](https://www.ncbi.nlm.nih.gov/pubmed/28611229)  62(10):1324-1332.
9. Singer M,Deutschman CS, Seymour CW, et al (2016) The Third International Consensus Definitions for Sepsis and Septic Shock (Sepsis-3). *JAMA*. 315(8):801-810.
10. [Dellinger RP](https://www.ncbi.nlm.nih.gov/pubmed/?term=Dellinger%20RP%5BAuthor%5D&cauthor=true&cauthor_uid=23353941), [Levy MM](https://www.ncbi.nlm.nih.gov/pubmed/?term=Levy%20MM%5BAuthor%5D&cauthor=true&cauthor_uid=23353941), [Rhodes A](https://www.ncbi.nlm.nih.gov/pubmed/?term=Rhodes%20A%5BAuthor%5D&cauthor=true&cauthor_uid=23353941), et al (2013) Surviving sepsis campaign: international guidelines for management of severe sepsis and septic shock, 2012. [*Crit Care Med.*](https://www.ncbi.nlm.nih.gov/pubmed/23353941)  41(2):580-637.
11. HerdmanM, Gudex C, Lloyd A, Janssen M, Kind P, Parkin D, Bonsel G, Badia X (2011) Development and preliminary testing of the new five-level version of EQ-5D (EQ-5D-5L). *Qual Life Res.* 20:1727-1736.
12. Patrick DL, Erickson P: Health Status And Health Policy. Oxford University Press: New York, NY; 1993
13. Dolan P (1997) Modeling valuations for EuroQol healthstates. *Med Care.* 35:1095-1108.
14. Vainiola T, Roine RP,Pettilä V, Kantola T, Räsänen P, Sintonen H (2011) Effect of health-related quality-of-life instrument and quality-adjusted life year calculation method on the number of life years gained in the critical care setting. *Value Health*. 14:1130-1134.
15. vanHout B, Janssen MF,  [Feng YS](https://www.ncbi.nlm.nih.gov/pubmed/?term=Feng%20YS%5BAuthor%5D&cauthor=true&cauthor_uid=22867780), [Kohlmann T](https://www.ncbi.nlm.nih.gov/pubmed/?term=Kohlmann%20T%5BAuthor%5D&cauthor=true&cauthor_uid=22867780), [Busschbach J](https://www.ncbi.nlm.nih.gov/pubmed/?term=Busschbach%20J%5BAuthor%5D&cauthor=true&cauthor_uid=22867780), [Golicki D](https://www.ncbi.nlm.nih.gov/pubmed/?term=Golicki%20D%5BAuthor%5D&cauthor=true&cauthor_uid=22867780), [Lloyd A](https://www.ncbi.nlm.nih.gov/pubmed/?term=Lloyd%20A%5BAuthor%5D&cauthor=true&cauthor_uid=22867780), [Scalone L](https://www.ncbi.nlm.nih.gov/pubmed/?term=Scalone%20L%5BAuthor%5D&cauthor=true&cauthor_uid=22867780), [Kind P](https://www.ncbi.nlm.nih.gov/pubmed/?term=Kind%20P%5BAuthor%5D&cauthor=true&cauthor_uid=22867780), [Pickard AS](https://www.ncbi.nlm.nih.gov/pubmed/?term=Pickard%20AS%5BAuthor%5D&cauthor=true&cauthor_uid=22867780) (2012) Interim scoring for the EQ-5D-5L: mapping the EQ-5D-5L to EQ-5D-3L value sets. *Value Health.* 15(5):708-715.
16. Horan TC, Andrus M, Dudeck MA (2008) CDC/NHSN surveillance deﬁnition of healthcare-associated infection and criteria for speciﬁc types of infections in the acutecaresetting. *Am J InfectControl.* 36:309-332.
17. Hugonnet S, Sax H, Eggimann P, Chevrolet JC, Pittet D (2004) Nosocomial bloodstream infection and clinical sepsis. *Emerg Infect Dis*. 10:76-81.
18. Maki DG, Weise CE, Sarafin HW (1977) A semiquantitative culturemethodforidentifyingintravenous-caheter-relatedinfection. *N Engl J Med.*  296:1305–1309.
19. Brun-Buisson C, Abrouk F, Legrand P, Huet Y, Larabi S, Rapin M (1987) Diagnosis ofcentralvenouscatheter-relatedsepsis. Critical levelof quantitative tip cultures. *Arch Intern Med*.  147:873–877.
20. [Nseir S](https://www.ncbi.nlm.nih.gov/pubmed/?term=Nseir%20S%5BAuthor%5D&cauthor=true&cauthor_uid=12503708), [Di Pompeo C](https://www.ncbi.nlm.nih.gov/pubmed/?term=Di%20Pompeo%20C%5BAuthor%5D&cauthor=true&cauthor_uid=12503708), [Pronnier P](https://www.ncbi.nlm.nih.gov/pubmed/?term=Pronnier%20P%5BAuthor%5D&cauthor=true&cauthor_uid=12503708), [Beague S](https://www.ncbi.nlm.nih.gov/pubmed/?term=Beague%20S%5BAuthor%5D&cauthor=true&cauthor_uid=12503708), [Onimus T](https://www.ncbi.nlm.nih.gov/pubmed/?term=Onimus%20T%5BAuthor%5D&cauthor=true&cauthor_uid=12503708), [Saulnier F](https://www.ncbi.nlm.nih.gov/pubmed/?term=Saulnier%20F%5BAuthor%5D&cauthor=true&cauthor_uid=12503708), [Grandbastien B](https://www.ncbi.nlm.nih.gov/pubmed/?term=Grandbastien%20B%5BAuthor%5D&cauthor=true&cauthor_uid=12503708), [Mathieu D](https://www.ncbi.nlm.nih.gov/pubmed/?term=Mathieu%20D%5BAuthor%5D&cauthor=true&cauthor_uid=12503708), [Delvallez-Roussel M](https://www.ncbi.nlm.nih.gov/pubmed/?term=Delvallez-Roussel%20M%5BAuthor%5D&cauthor=true&cauthor_uid=12503708), [Durocher A](https://www.ncbi.nlm.nih.gov/pubmed/?term=Durocher%20A%5BAuthor%5D&cauthor=true&cauthor_uid=12503708) (2002) Nosocomial tracheobronchitis in mechanically ventilated patients: incidence, aetiology and outcome. [*EurRespir J.*](https://www.ncbi.nlm.nih.gov/pubmed/12503708)  20(6):1483-1489.
21. [Armaganidis A](https://www.ncbi.nlm.nih.gov/pubmed/?term=Armaganidis%20A%5BAuthor%5D&cauthor=true&cauthor_uid=28425571), [Nanas S](https://www.ncbi.nlm.nih.gov/pubmed/?term=Nanas%20S%5BAuthor%5D&cauthor=true&cauthor_uid=28425571), [Antoniadou E](https://www.ncbi.nlm.nih.gov/pubmed/?term=Antoniadou%20E%5BAuthor%5D&cauthor=true&cauthor_uid=28425571), [Mandragos K](https://www.ncbi.nlm.nih.gov/pubmed/?term=Mandragos%20K%5BAuthor%5D&cauthor=true&cauthor_uid=28425571), [Liakou K](https://www.ncbi.nlm.nih.gov/pubmed/?term=Liakou%20K%5BAuthor%5D&cauthor=true&cauthor_uid=28425571), [Koutsoukou A](https://www.ncbi.nlm.nih.gov/pubmed/?term=Koutsoukou%20A%5BAuthor%5D&cauthor=true&cauthor_uid=28425571), [Baltopoulos G](https://www.ncbi.nlm.nih.gov/pubmed/?term=Baltopoulos%20G%5BAuthor%5D&cauthor=true&cauthor_uid=28425571), [Nakos G](https://www.ncbi.nlm.nih.gov/pubmed/?term=Nakos%20G%5BAuthor%5D&cauthor=true&cauthor_uid=28425571), [Kounougeri A](https://www.ncbi.nlm.nih.gov/pubmed/?term=Kounougeri%20A%5BAuthor%5D&cauthor=true&cauthor_uid=28425571), [Ganas K](https://www.ncbi.nlm.nih.gov/pubmed/?term=Ganas%20K%5BAuthor%5D&cauthor=true&cauthor_uid=28425571), [Prekates A](https://www.ncbi.nlm.nih.gov/pubmed/?term=Prekates%20A%5BAuthor%5D&cauthor=true&cauthor_uid=28425571), [Kompoti M](https://www.ncbi.nlm.nih.gov/pubmed/?term=Kompoti%20M%5BAuthor%5D&cauthor=true&cauthor_uid=28425571), [Georgopoulos D](https://www.ncbi.nlm.nih.gov/pubmed/?term=Georgopoulos%20D%5BAuthor%5D&cauthor=true&cauthor_uid=28425571), [Pneumatikos I](https://www.ncbi.nlm.nih.gov/pubmed/?term=Pneumatikos%20I%5BAuthor%5D&cauthor=true&cauthor_uid=28425571), [Zakynthinos E](https://www.ncbi.nlm.nih.gov/pubmed/?term=Zakynthinos%20E%5BAuthor%5D&cauthor=true&cauthor_uid=28425571) (2017) Clinical factors affecting costs in patients receiving systemic antifungal therapy in intensive care units in Greece: Results from the ESTIMATOR study. [*Mycoses.*](https://www.ncbi.nlm.nih.gov/pubmed/28425571)  60(7):454-461.
22. Seidel J, Whiting PC, Edbrooke DL (2006) The costs of intensive care. *Continuing Education in Anaesthesia, Critical Care &Pain.* 6(4):160-163.

***Supplemental Table 1*.** Multivariate binary regression model examining the effect of univariate risk factors (Table 2; main text) for the development of ABRP infections in ICU. A 2-tailed P value <0.05 was considered to be significant.

|  | **OR** | **95% C.I. for OR** | | **P value** |
| --- | --- | --- | --- | --- |
| Cardiac arrest | 1.944 | 0.698 | 5.516 | 0.203 |
| APACHE II | 1.037 | 0.981 | 1.096 | 0.199 |
| ARDS | 1.274 | 0.572 | 2.837 | 0.553 |
| MODS score | 0.676 | 0.584 | 0.782 | **<0.001** |
| CRRT | 4.453 | 1.805 | 10.982 | **0.001** |
| ABRP= antibiotic resistant pathogen; ICU=intensive care unit; OR= odds ratio; CI= confidence interval; APACHE II= Acute Physiology and Chronic Health Evaluation Score II; ARDS= acute respiratory distress syndrome; MODS= multiple organ dysfunction syndrome; CRRT= continuous renal replacement therapy | | | | |

***Supplemental Table 2.*** Univariate and multivariate linear regression analysis for ICU cost. Only significant univariate determinants of ICU cost were included in the multivariate model.

|  |  | Univariate linear regression analysis |  |  |  | Multivariate linear regression analysis |  |
| --- | --- | --- | --- | --- | --- | --- | --- |
| Variables | *β* | Β (95% CI for B) | P |  | *β* | B (95% CI for B) | P |
| (Constant) | - | - | - |  | - | 3149.1 (1956.8 – 4341.4) | <0.001 |
| APACHE II | 0.154 | 133.6 (43.4 – 223.8) | 0.004 |  | 0.010 | 7.99 (-58.24 - 74.22) | 0.813 |
| Sepsis | 0.105 | 1397.5 (2.1-2792.9) | 0.05 |  | -0.025 | -336.8 (-1264.2 – 590.6) | 0.475 |
| Septic shock | 0.195 | 3047 (1430.6 – 4663.5) | <0.001 |  | -0.031 | -467.5 (-1806.6 – 871.6) | 0.493 |
| Cardiac arrest | 0.145 | 3131.5 (862.8 – 5400.2) | 0.007 |  | 0.074 | 1609.3 (158.8 – 3059.9) | **0.030** |
| ARDS | 0.277 | 4103.7 (2600.8 – 5606.6) | <0.001 |  | 0.035 | 504.1 (-638.2 – 1646.5) | 0.386 |
| MODS score | 0.251 | 600.4 (355.3 – 845.5) | <0.001 |  | -0.012 | -27.6 (-243.3 - 188) | 0.801 |
| Obesity | 0.118 | 2901.6 (321.1 – 5482.1) | 0.028 |  | 0.038 | 917.2 (-669.5 - 2504) | 0.256 |
| ARP | 0.518 | 8525.4 (7044.9 – 10005.9) | <0.001 |  | -0.073 | -1190.8 (-3778.7 - 1397) | 0.366 |
| BSI | 0.550 | 7438.8 (6248.7 – 8628.8) | <0.001 |  | 0.081 | 1071.8 (-271.8 – 2415.5) | 0.118 |
| BSI- ABRP | 0.503 | 8945 (7327.7 – 10562.3) | <0.001 |  | 0.148 | 2586.9 (124 – 5241.6) | **0.046** |
| Non-BSI | 0.422 | 6677.5 (5167.9 – 8187.2) | <0.001 |  | -0.005 | -11.4 (-182 – 159.12) | 0.895 |
| Colistin | 0.680 | 9173.6 (8130.3 – 10216.9) | <0.001 |  | 0.367 | 4824.4 (3578.8 - 6070.1) | **<0.001** |
| Tracheostomy | 0.597 | 11696.2 (10038 – 13354.4) | <0.001 |  | 0.335 | 6414.7 (4913.2 – 7916.4) | **<0.001** |
| Myopathy | 0.531 | 9995.6 (8317 – 11674.2) | <0.001 |  | 0.179 | 3271.1 (1848 – 4694.3) | **<0.001** |
| B,β, Unstandardized and standardized β coefficients, respectively; CI= confidence interval; APACHE II, Acute Physiology and Chronic Health Evaluation Score II; ARDS=acute respiratory distress syndrome; MODS= multi-organ dysfunction syndrome; ABRP= antibiotic-resistant pathogens; BSI= blood stream infections; BSI-ARP= blood stream infections due to ARPs; Non-BSI= other (except for BSI) sites of infections. | | | | | | | |

***Supplemental Table 3.*** Cox regression survival analysis examining the effect of MDR infections on 2-year mortality, after adjusting for possible covariates of severe underlying illness.

|  |  | **95% CI** | |  |
| --- | --- | --- | --- | --- |
|  | *HR* | *Lower* | *Upper* | *P value* |
| **APACHE II** | 1.102 | 1.077 | 1.126 | **<0.001** |
| **MODS score** | 1.103 | 1.050 | 1.157 | **<0.001** |
| **ARDS** | 0.799 | 0.561 | 1.138 | 0.214 |
| **CRRT** | 1.341 | 0.834 | 2.156 | 0.226 |
| **MV prior to infection** | 0.754 | 0.501 | 1.133 | 0.174 |
| **Cardiac arrest** | 1.122 | 0.698 | 1.802 | 0.635 |
| **MDR infections** | 1.300 | 0.824 | 2.050 | 0.259 |
| MDR= multi-drug resistant pathogens; CI= confidence interval; HR= hazard ratio; APACHE II= Acute Physiology and Chronic Health Evaluation Score II; MODS= multiple organ dysfunction syndrome; ARDS= acute respiratory distress syndrome; CRRT= continuous renal replacement therapy; MV= mechanical ventilation.  Variables included in the Models were assessed for multicolinearity issues; All Variable Inflation Factors (VIF) ≤1.796 | | | | |
|  | | | | |

***Supplemental Table 4.*** *M*ultivariate linear regression analysis examining the effect of infections due to ABRP (Model 1) and XDR organisms (Model 2) on 2-year QALY values, adjusting for putative variables of critical illness severity.

|  | **Multivariate linear regression analysis** | | | | | | |
| --- | --- | --- | --- | --- | --- | --- | --- |
|  | ***Model 1*** | | |  | ***Model 2*** | | |
| **Variables** | *β* | Β (95% CI for B) | P |  | *β* | B (95% CI for B) | P |
| **(Constant)** | - | 1.153 (0.751, 1.544) | **<0.001** |  | - | 1.145 (0.746, 1.545) | **<0.001** |
| **APACHE II** | -0.189 | -0.030 (-0.049, -0.010) | **0.003** |  | -0.181 | -0.029 (-0.048, -0.009) | **0.004** |
| **MODS score** | -0.097 | -0.042 (-0.102, 0.018) | 0.166 |  | -0.119 | -0.052 (-0.111, 0.007) | 0.086 |
| **ARDS** | -0.005 | -0.013 (-0.327, 0.302) | 0.937 |  | -0.008 | -0.021 (-0.335, 0.293) | 0.897 |
| **CRRT** | 0.066 | 0.253 (-0.164, 0.669) | 0.233 |  | 0.061 | 0234 (-0.177, 0.644) | 0.263 |
| **MV** | -0.047 | -0.133 (-0.435, 0.170) | 0.389 |  | -0.051 | -0.144 (-0.447, 0.159) | 0.349 |
| **Cardiac arrest** | 0.059 | 0.243 (-0.199, 0.684) | 0.280 |  | 0.055 | 0.224 (-0.218, 0.666) | 0.319 |
| **ABRP infections** | -0.003 | -0.009 (-0.367, 0.348) | 0.960 |  | - | - | - |
| **XDR infections** | - | - | - |  | 0.049 | 0.220 (-0.303, 0.743) | 0.409 |
| ABRP= antibiotic resistant pathogens; XDR= extensively-drug resistant pathogens; B,β= Unstandardized and standardized β coefficients, respectively; CI= confidence interval; APACHE II= Acute Physiology and Chronic Health Evaluation Score II; MODS= multi-organ dysfunction syndrome; ARDS=acute respiratory distress syndrome; CRRT= continuous renal replacement therapy; MV= mechanical ventilation prior to ICU infection.  Variables included in the Models were assessed for multi-colinearity issues; All Variable Inflation Factors (VIF) ≤1.796 | | | | | | | |
