# Supplementary figures and images for: The role of antimicrobial resistance on long-term mortality and quality of life in critically ill patients: a prospective longitudinal 2-year study
Source: Health Qual Life Outcomes. 2021 Mar 3;19:72. doi: 10.1186/s12955-021-01712-0 (PMC7927260; doi:10.1186/s12955-021-01712-0)

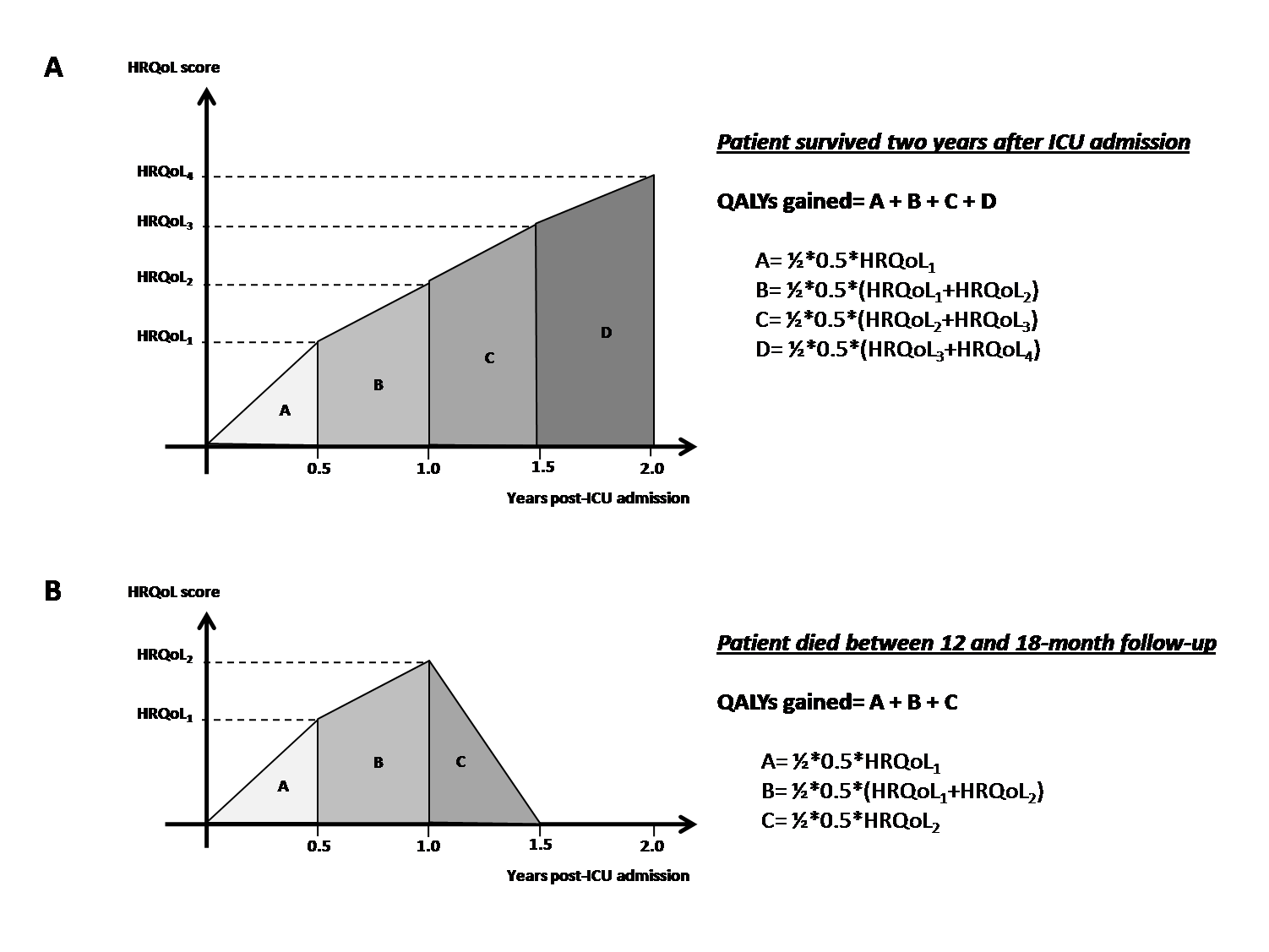

Supplement: Supplementary file 2 — Additional file 2: Supplemental Figure 1 Examples of calculation of QALY-gained in our study. A. QALYs-gained by ICU treatment in a patient having survived the 2-year follow-up (QALYs gained= area A + area B + area C + area D). B. QALYs-gained by ICU treatment in the theoretical patient who died between the 12-month and the 18-month follow-up (QALYs gained= area A+ area B + area C). HRQoL= health-related quality of life; HRQoL1,2,3,4= utility index values at 6,12,18 and 24-month follow-up, respectively; ICU= intensive care unit; QALYs= quality-adjusted life years. [file 12955_2021_1712_MOESM2_ESM.tif]

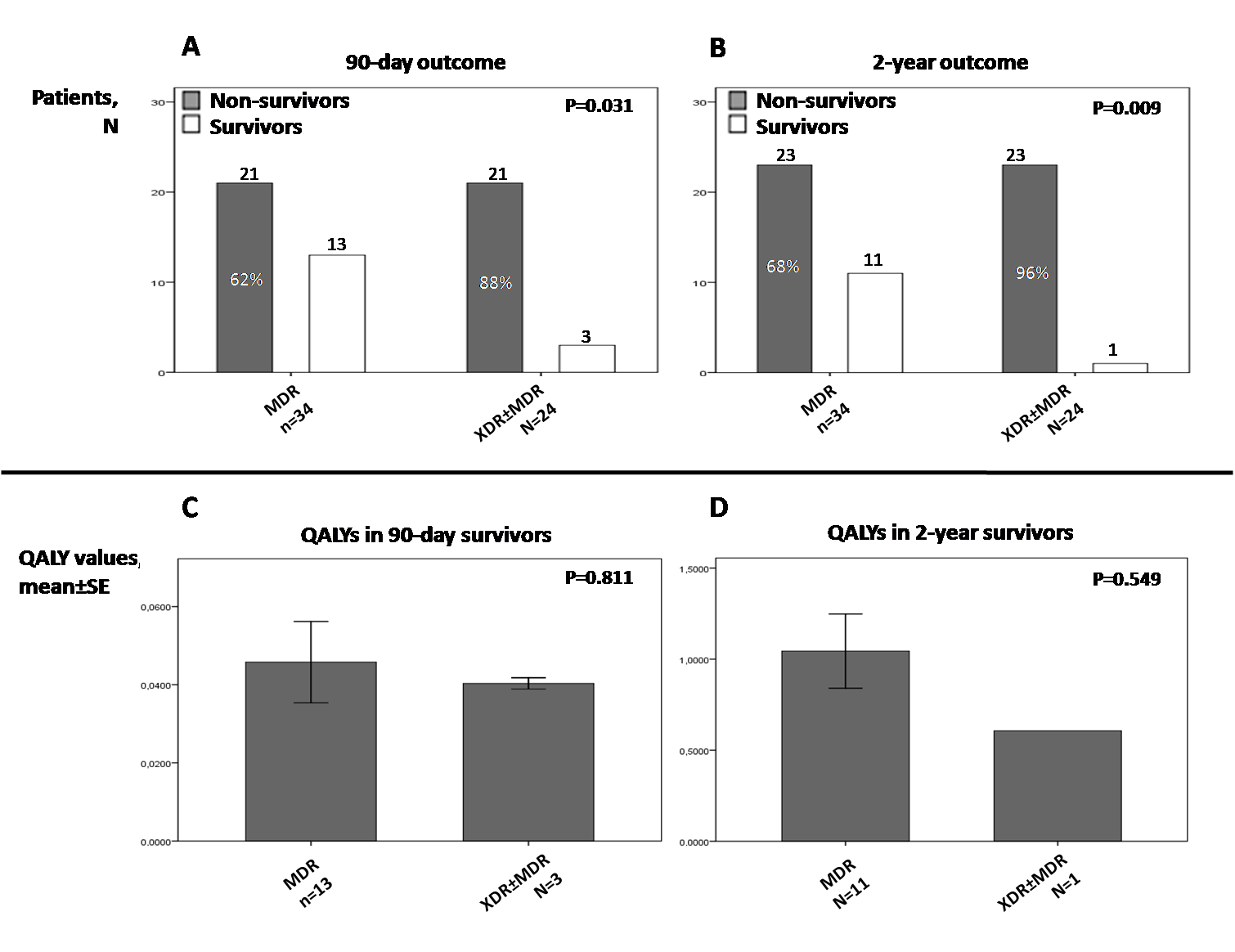

Supplement: Supplementary file 3 — Additional file 3: Supplemental Figure 2. Long-term outcomes in the MDR/XDR subgroups of our 58 patients with antibiotic resistant pathogens. In the upper panel, bars represent the number of 90-day (A) and 2-year (B) survivors and non-survivors with MDR and XDR infections; percentages within grey bars represent mortality in each category. In the lower panel, bars and vertical lines indicate mean QALY values and standard errors in 90-day and 2-year survivors, respectively. MDR= multi-drug resistant pathogen; XDR= extensively-drug resistant pathogen; QALY= quality-adjusted life years. [file 12955_2021_1712_MOESM3_ESM.tif]

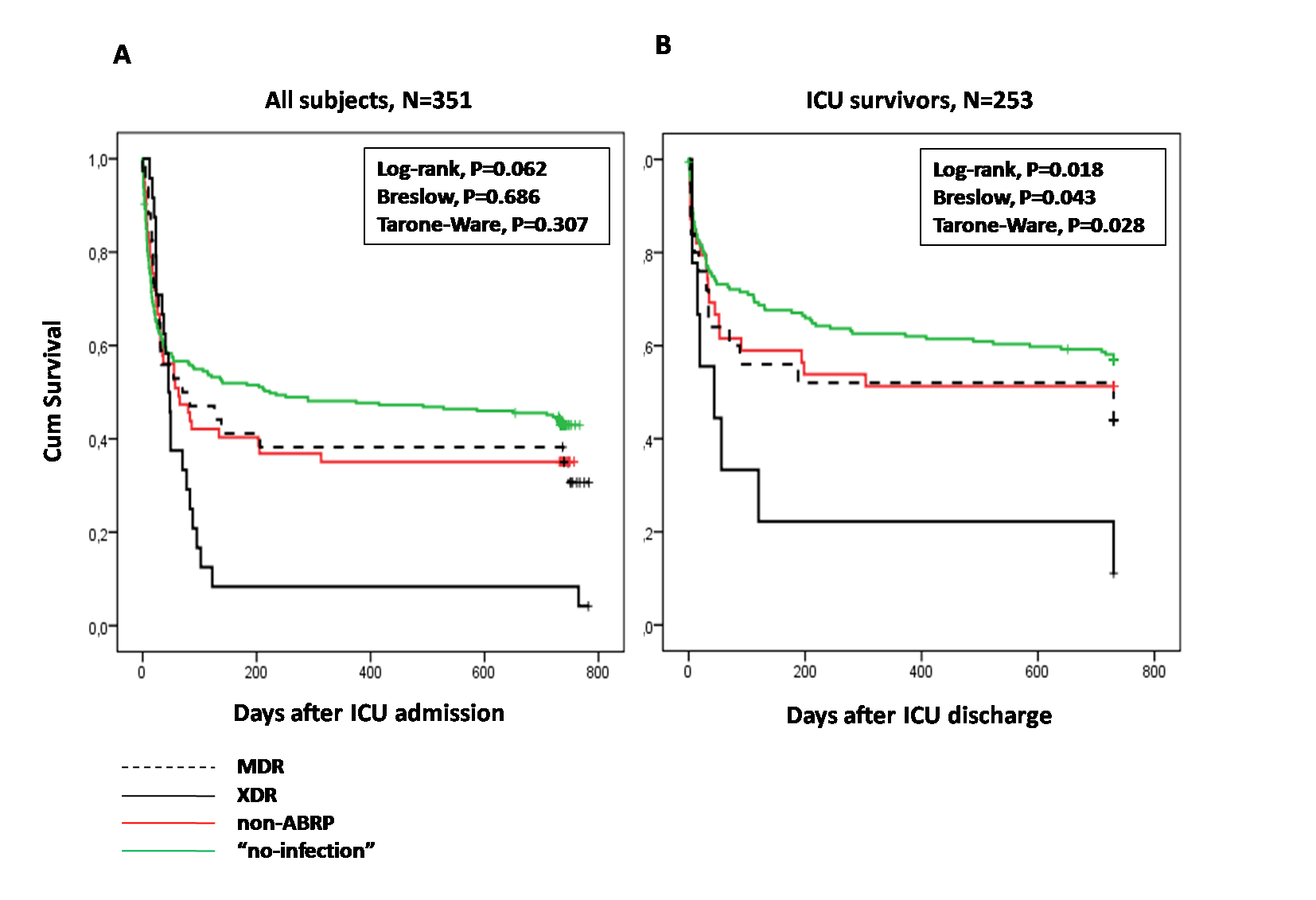

Supplement: Supplementary file 4 — Additional file 4: Supplemental Figure 3. Kaplan-Meier 2-year survival curves examining the effect of ABRP infections on long-term mortality post-discharge. Analysis was performed in our 351 ICU patients overall (panel A), and in the subset of 253 ICU survivors (panel B). Patients were divided into four groups according to having demonstrated MDR infection(s) (black dotted line), XDR infection(s) (continuous black line), infections due to non-ABRPs (red line) or “no-infection” at all (green line) while in ICU. ABRP= antibiotic resistant pathogen; MDR= multi-drug resistant; XDR= extensively-drug resistant; ICU= intensive care unit. [file 12955_2021_1712_MOESM4_ESM.tif]

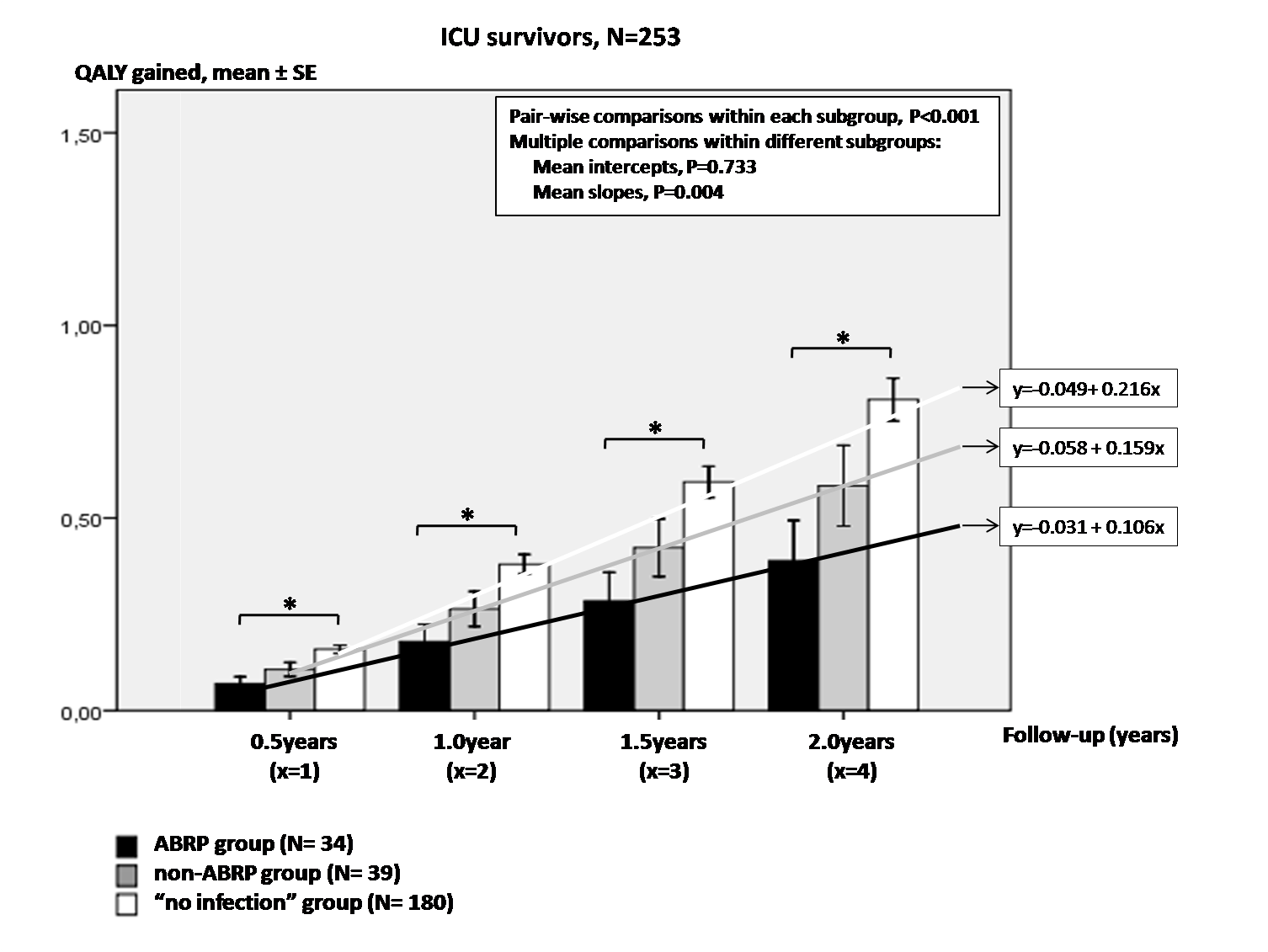

Supplement: Supplementary file 5 — Additional file 5: Supplemental Figure 4. Two-year QALY kinetics in ABRP, non-ABRP and “no-infection” subgroups of our ICU survivors (N=253). Bars and vertical lines indicate mean QALY values and standard errors, respectively. ABRP group demonstrates lower increase in QALYs over time (markedly depressed slope of the corresponding mean regression line) compared to its counterparts. QALY= quality-adjusted life years; ABRP= antibiotic resistant pathogen; ICU= intensive care unit; SE= standard error; Intercept of the regression line= the QALY value where the regression line crosses the y-axis at the theoretical day=0; Slope of the regression line= the rate at which QALY values change between two-consequent follow-up examinations. *P<0.001. [file 12955_2021_1712_MOESM5_ESM.tif]

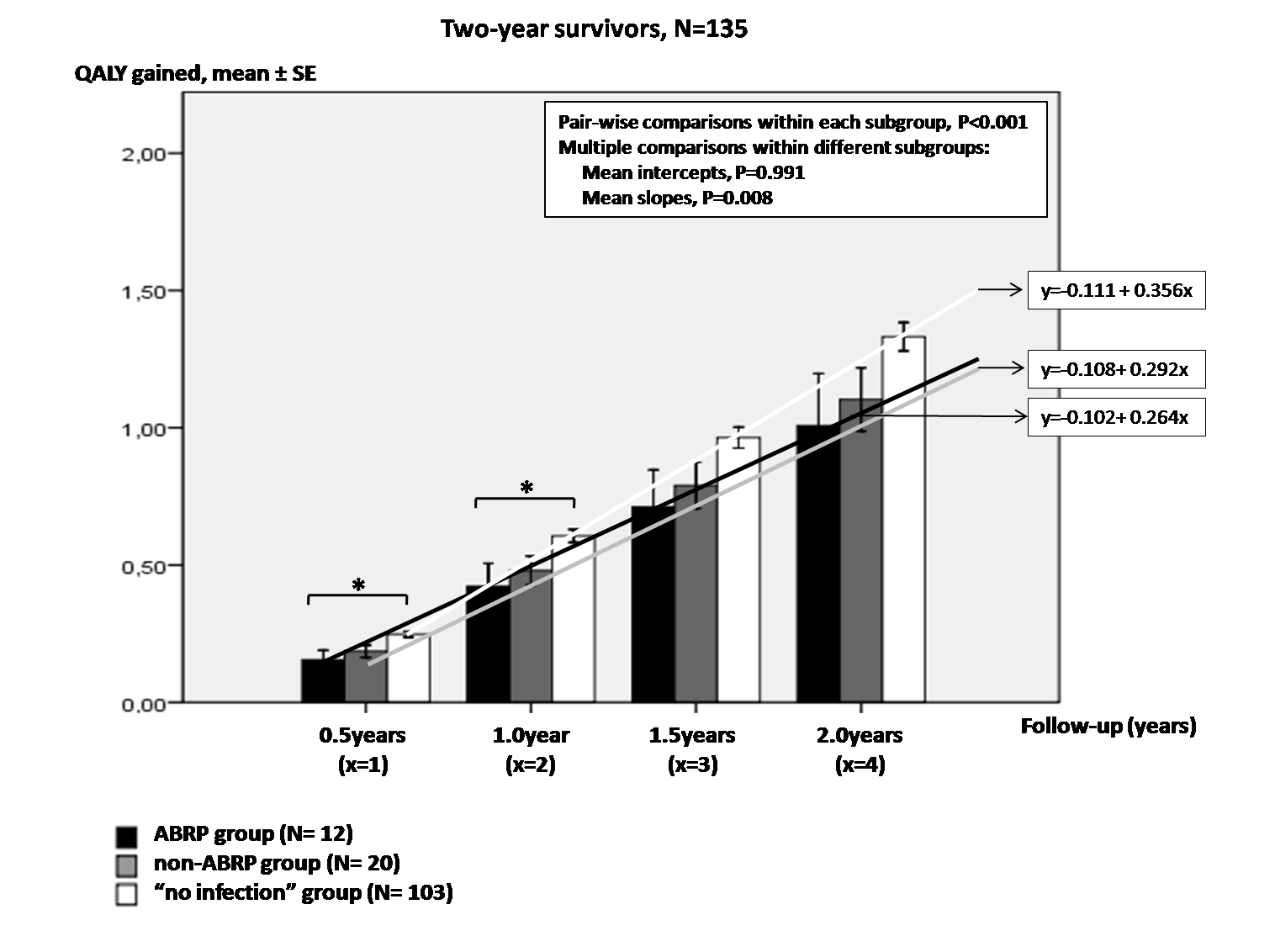

Supplement: Supplementary file 6 — Additional file 6: Supplemental Figure 5. Two-year QALY kinetics in ABRP, non-ABRP and “no-infection” subgroups of our 2-year survivors (N=135). Bars and vertical lines indicate mean QALY values and standard errors, respectively. ABRP group demonstrates lower increase in QALYs over time (lower slope of the corresponding mean regression line) compared to its counterparts. QALY= quality-adjusted life years; ABRP= antibiotic resistant pathogen; SE= standard error; Intercept of the regression line= the QALY value where the regression line crosses the y-axis at the theoretical day=0; Slope of the regression line= the rate at which QALY values change between two-consequent follow-up examinations.*P<0.05. [file 12955_2021_1712_MOESM6_ESM.tif]

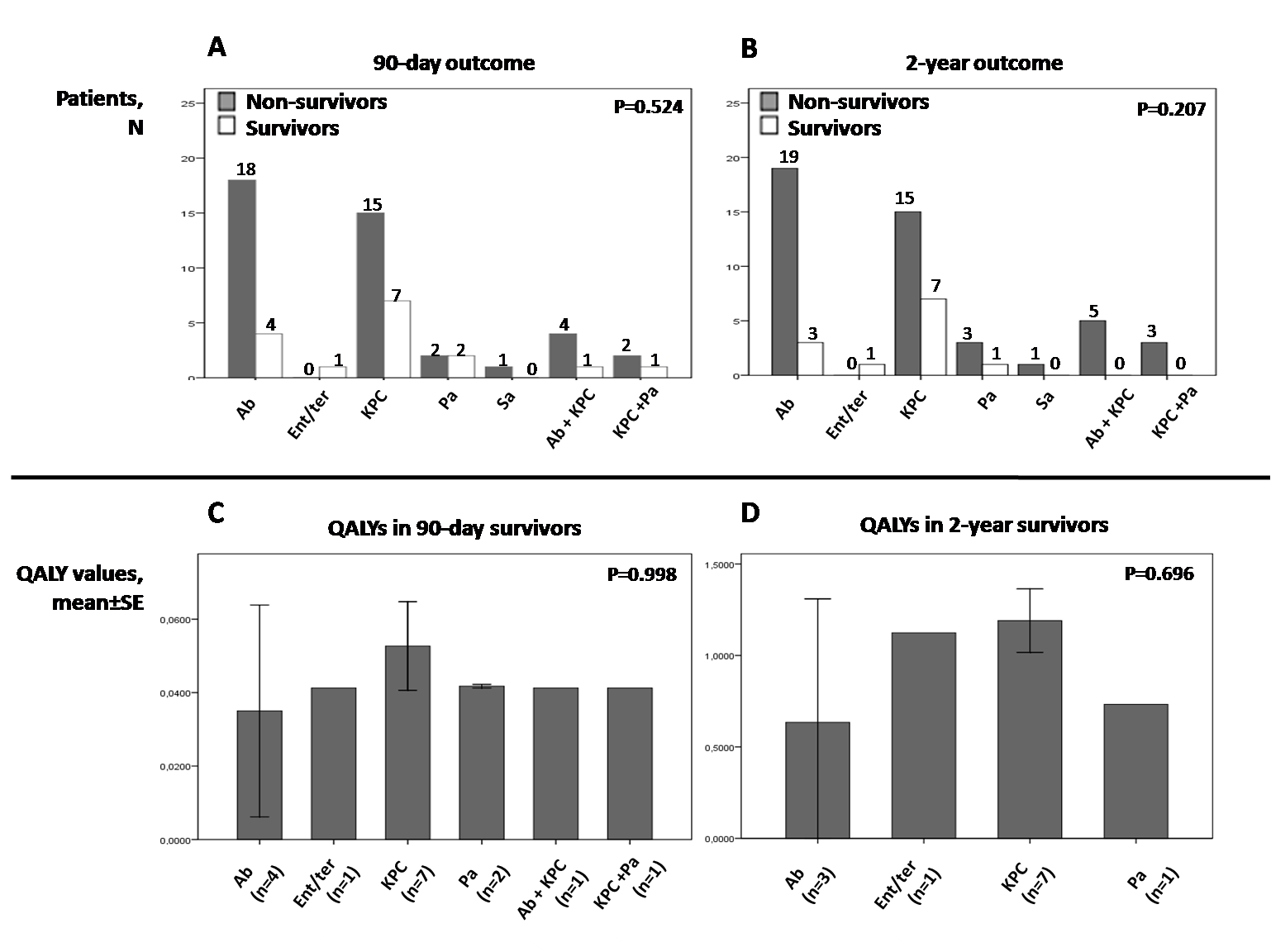

Supplement: Supplementary file 7 — Additional file 7: Supplemental Figure 6. Long-term outcomes in our 58 ABRP patients according to underlying pathogen. In the upper panel, bars represent number of 90-day (A) and 2-year (B) survivors and non-survivors with respect to underlying pathogen(s). In the lower panel, bars and vertical lines indicate mean QALY values and standard errors in 90-day and 2-year survivors regarding the underlying pathogen(s), respectively. ABRP= antibiotic resistant pathogens; QALY= quality-adjusted life years; Ab=Acinetobacter baumannii; Ent/ter=Enterobacter cloacae; KPC= Klebsiella pneumonia producing carbapenemases; Pa= Pseudomonas aeruginosa; Sa= staphylococcus aureus. [file 12955_2021_1712_MOESM7_ESM.tif]

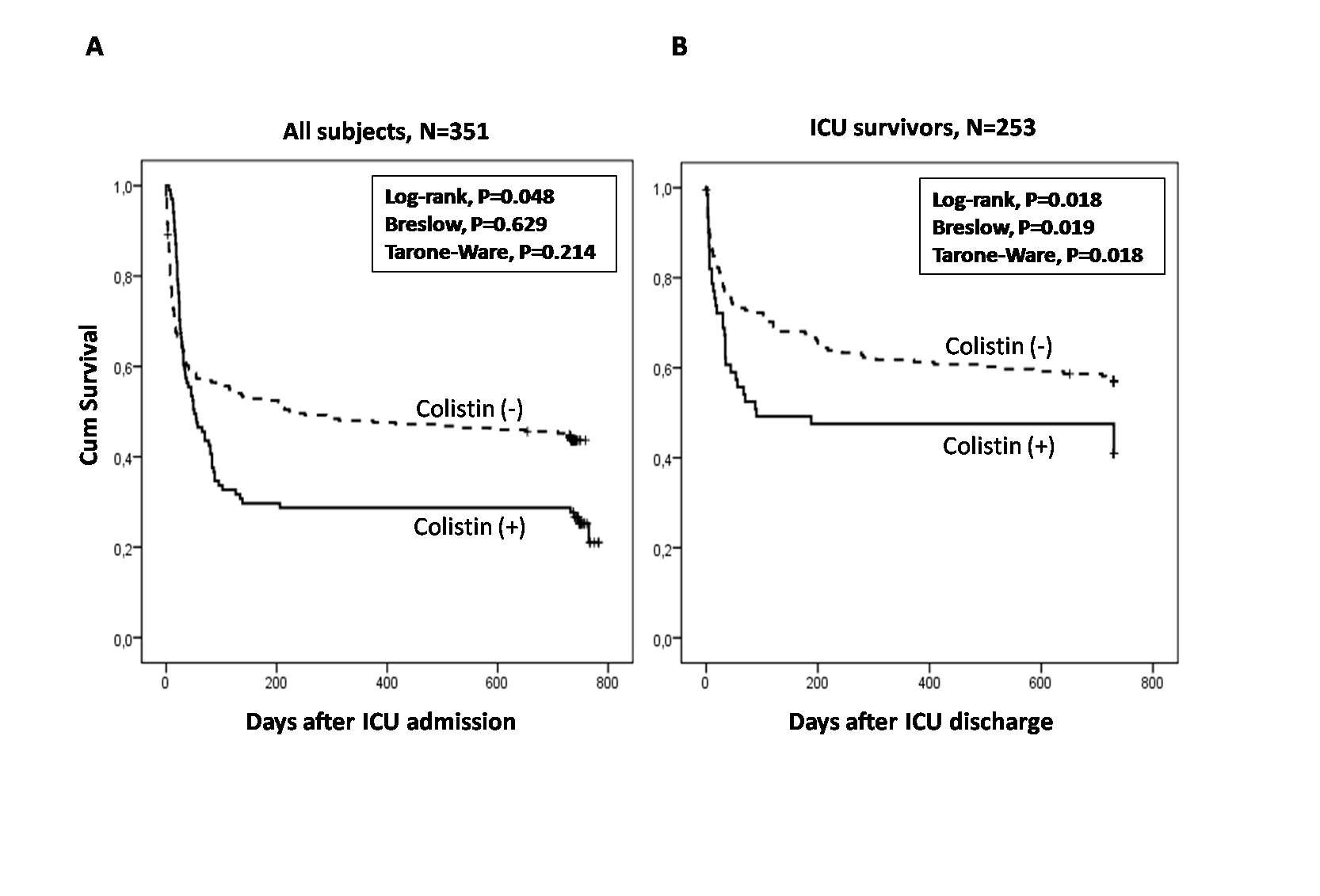

Supplement: Supplementary file 8 — Additional file 8: Supplemental Figure 7. Kaplan-Meier survival curves examining the effect of colistin administration in ICU on long-term mortality post-discharge. Analysis was performed in our 351 ICU patients overall (panel A), and in the subset of 253 ICU survivors (panel B). ICU= intensive care unit. [file 12955_2021_1712_MOESM8_ESM.tif]

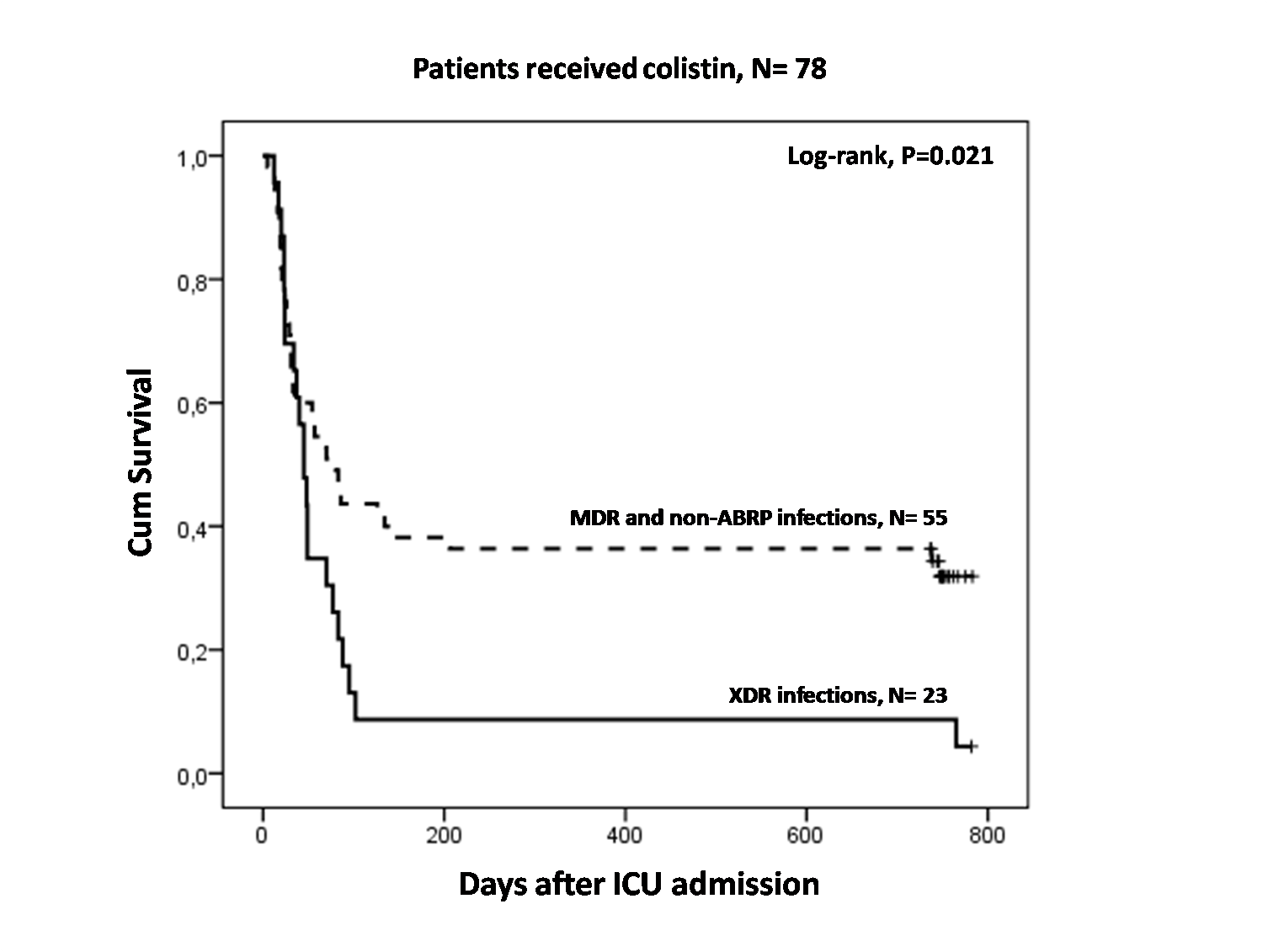

Supplement: Supplementary file 9 — Additional file 9: Supplemental Figure 8. Kaplan-Meier survival curves examining the effect of XDR versus non-XDR (MDR plus non-ABRP) infections on 2-year mortality in our 78 patients treated with colistin. XDR= extensively-drug resistant pathogens; MDR= multidrug resistant pathogens; ABRP= antibiotic resistant pathogens. [file 12955_2021_1712_MOESM9_ESM.tif]
